# Kv2 channels do not function as canonical delayed rectifiers in spinal motoneurons

**DOI:** 10.1016/j.isci.2024.110444

**Published:** 2024-07-03

**Authors:** Calvin C. Smith, Filipe Nascimento, M. Görkem Özyurt, Marco Beato, Robert M. Brownstone

**Affiliations:** 1 Department of Neuromuscular Diseases, UCL Queen Square Institute of Neurology, University College London, London WC1N 3BG, UK; 2 Department of Neuroscience, Physiology, and Pharmacology, University College London, London WC1E 6BT, UK

**Keywords:** Neuroscience, Behavioral neuroscience, Sensory neuroscience

## Abstract

The increased muscular force output required for some behaviors is achieved via amplification of motoneuron output via cholinergic C-bouton synapses. Work in neonatal mouse motoneurons suggested that modulation of currents mediated by post-synaptically clustered K_V_2.1 channels is crucial to C-bouton amplification. By focusing on more mature motoneurons, we show that conditional knockout of K_V_2.1 channels minimally affects either excitability or response to exogenously applied muscarine. Similarly, unlike in neonatal motoneurons or cortical pyramidal neurons, pharmacological blockade of K_V_2 currents has minimal effect on mature motoneuron firing *in vitro*. Furthermore, *in vivo* amplification of electromyography activity and high-force task performance was unchanged following K_V_2.1 knockout. Finally, we show that K_V_2.2 is also expressed by spinal motoneurons, colocalizing with K_V_2.1 opposite C-boutons. We suggest that the primary function of K_V_2 proteins in motoneurons is non-conducting and that K_V_2.2 can function in this role in the absence of K_V_2.1.

## Highlights

• K_V_2 channels do not regulate firing characteristics in mature motoneurons
• Firing and muscarinic amplification are preserved in ChAT-K_V_2.1 knockout mice
• Behavior and motor amplification are preserved in ChAT-K_V_2.1 knockout mice
• With K_V_2.2, motoneuron K_V_2.1 channels likely play primarily structural roles

## Graphical abstract

**Figure:**
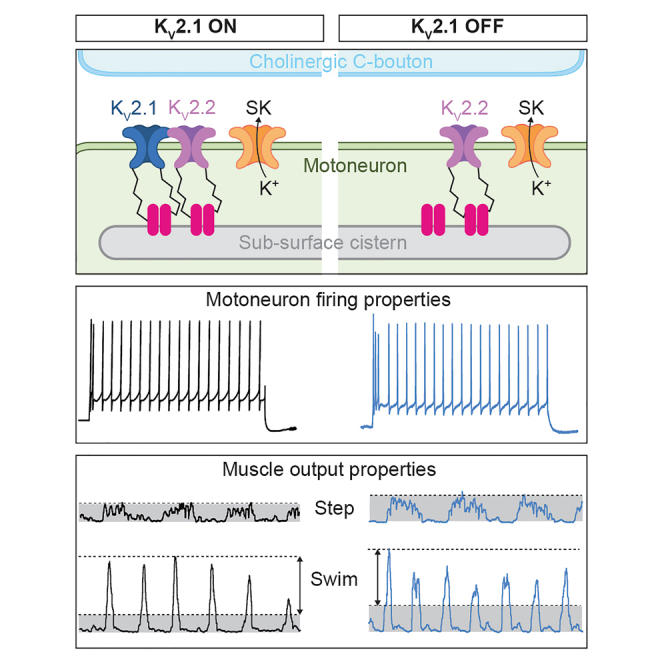

## Introduction

Animals can produce a vast repertoire of behaviors by altering spatiotemporal patterns of muscle contractions, which are governed by the motoneurons that innervate them. Therefore, motoneuron activity must be regulated so as to support diverse motor outputs. In addition to muscle fiber type properties, the strength of muscle contraction is governed by the number of active innervating motoneurons and their firing frequencies.^1^ Thus, to understand the neural mechanisms of behavior, it is essential to understand how motoneurons produce repetitive spike trains.

The relationship between the inputs a motoneuron receives and its outputs is often represented by a “frequency-current” or ƒ-I curve, with the current being that injected through a recording electrode. Motoneuronal excitability, represented by the slope(s) of the linear segment(s) (gain) of this curve, can be altered in a task-dependent manner by neuromodulator systems. One system that can increase the gain is that comprised of presynaptic C-boutons, named because of their association with specialized endoplasmic reticulum (ER) called “subsurface cisterns”.^2^ These boutons arise from cholinergic V0c interneurons.^3^^,^^4^ The postsynaptic motoneuron membrane apposing C-boutons includes clusters of many proteins, including type 2 muscarinic acetylcholine (M2) receptors,^5^ slow calcium-dependent potassium channels (SK2, SK3)^6^ and voltage-gated delayed rectifier potassium channels (K_V_2.1).^7^ These synapses mature over the first three weeks of post-natal development in mice in parallel with the development of weight-bearing locomotor function.^8^ And in the adult mouse, this system is recruited for high force outputs such as the extensor stroke in swimming.^4^ But how activation of M2 receptors leads to the increase in excitability needed for these tasks remains elusive, leaving a significant hole in our understanding of movement.

It has been hypothesized that M2 receptor activation affects K_V_2.1 function to actuate C-bouton-mediated amplification.^7^^,^^9^^,^^10^ K_V_2 channels are widely expressed through the central nervous system.^11^ There are two main K_V_2 subunits, *Kcnb1* (K_V_2.1) and *Kcnb2* (K_V_2.2), which share similar biophysical properties, and are often, but not always, co-expressed.^12^^,^^13^^,^^14^^,^^15^ The canonical function of K_V_2 channels is to regulate neuronal excitability through delayed rectifier K^+^ currents.^16^ Their importance in neuronal function is evident by clinical reports of people with *Kcnb1* mutations, who have a myriad of problems including reduced cognitive capacity and epilepsy.^17^^,^^18^ About one-half of the 26 reported people are hypotonic, with 2/3 of these people having signs in early life. But mice with *Kcnb1* deletions are hyperactive; their problems are not in motoneuron function *per se*, and they are not hypotonic.^19^ These results do not support the hypothesis that K_V_2.1 channels are required for motor output. And they do not shed light on what the role of these channels in motoneurons might be.

Using K_V_2 (K_V_2.1 and K_V_2.2) channel blockers, several groups have suggested significant roles for K_V_2.1 conductances in C-bouton-mediated amplification in neonatal rodent motoneurons.^20^^,^^21^^,^^22^ Although results were not all entirely consistent between the studies, overall they suggested that when active, C-boutons recruit local K_V_2.1 channels to maintain narrow spikes and fast afterhyperpolarization (fAHP) amplitudes, thus supporting high-frequency firing by preventing Na^+^ channel inactivation and the resultant depolarization block. That is, current work suggests K_V_2.1 channels have significant conducting roles.

In motoneurons, K_V_2.1 channels at C-bouton synapses are densely clustered. In other neurons, this clustering results from a proximal restriction and clustering (PRC) domain, which was added to the C-terminal tail of K_V_2 during evolution.^16^^,^^23^ Although K_V_2 channels in this configuration are non-conducting,^24^^,^^25^ spatial aggregation of Kv2 via the PRC domain does not regulate conductance *per se*, because PRC mutations that cause de-clustering do not increase K_V_2 conductance.^26^^,^^27^ The K_V_2 PRC domain confers a structural role by binding to VAPs (vesicle associated membrane protein (VAMP) associated proteins) located on the endoplasmic reticulum (ER) membrane (EM), physically linking the plasma membrane (PM) to within 10 nm.^28^^,^^29^^,^^30^ Recent work has revealed that the tight ER-PM junctions (EPJs) conferred by Kv2 are essential for the spatial and functional coupling of several local Ca^2+^ signaling mechanisms crucial to cellular physiology.^27^^,^^31^^,^^32^ For example, in hippocampal neurons, K_V_2.1 promotes spatial and functional coupling of L-type calcium channels and ryanodine receptors (RyR) to mediate local calcium sparks.^33^ Although RyR have not been identified in motoneurons, given the calcium dependence of proteins clustered at C-boutons and the recent discovery of VAP expression in C-bouton post-synaptic domains,^9^ it is plausible that these dense clusters of K_V_2.1 proteins could serve similar non-conducting roles in motoneurons.

Taken together, the role of these prominent K_V_2.1 channels in regulating motoneuron firing and in C-bouton modulation remains unclear. We therefore aimed to define K_V_2.1 function in mature motoneurons from electrophysiology through to animal behavior. We made a conditional knock-out (cKO) mouse in which cholinergic neurons (including motoneurons) lacked K_V_2.1 channels. We then used whole-cell patch clamp electrophysiology to compare the firing characteristics of mature K_V_2.1^ON^ (control) and ChAT-K_V_2.1^OFF^ motoneurons and the effects of the specific K_V_2 channel blocker guangxitoxin-1E (GxTX-1E) on these properties. We repeated these experiments in early post-natal control motoneurons to assess whether developmental clustering of K_V_2.1 channels influences their role in regulating firing. To determine the role of K_V_2.1 in C-bouton function, we activated M2 receptors *in vitro* using muscarine and compared excitability changes in control and K_V_2.1 cKO motoneurons. Finally, to assess whether K_V_2.1 influences motor amplification, we studied high-force output behaviors while recording electromyography (EMG) activity in hindlimb muscles. In summary, we found that K_V_2.1 does not regulate mature motoneuron physiology or behavior, and suggest that K_V_2 channels primarily play a non-conducting role. Furthermore, we show that motoneurons also express K_V_2.2, suggesting it can subsume the non-conducting roles of K_V_2 channels to maintain C-bouton function.

## Results

### cKO of K_V_2.1 does not alter motoneuron passive membrane properties

**Figure 1:**
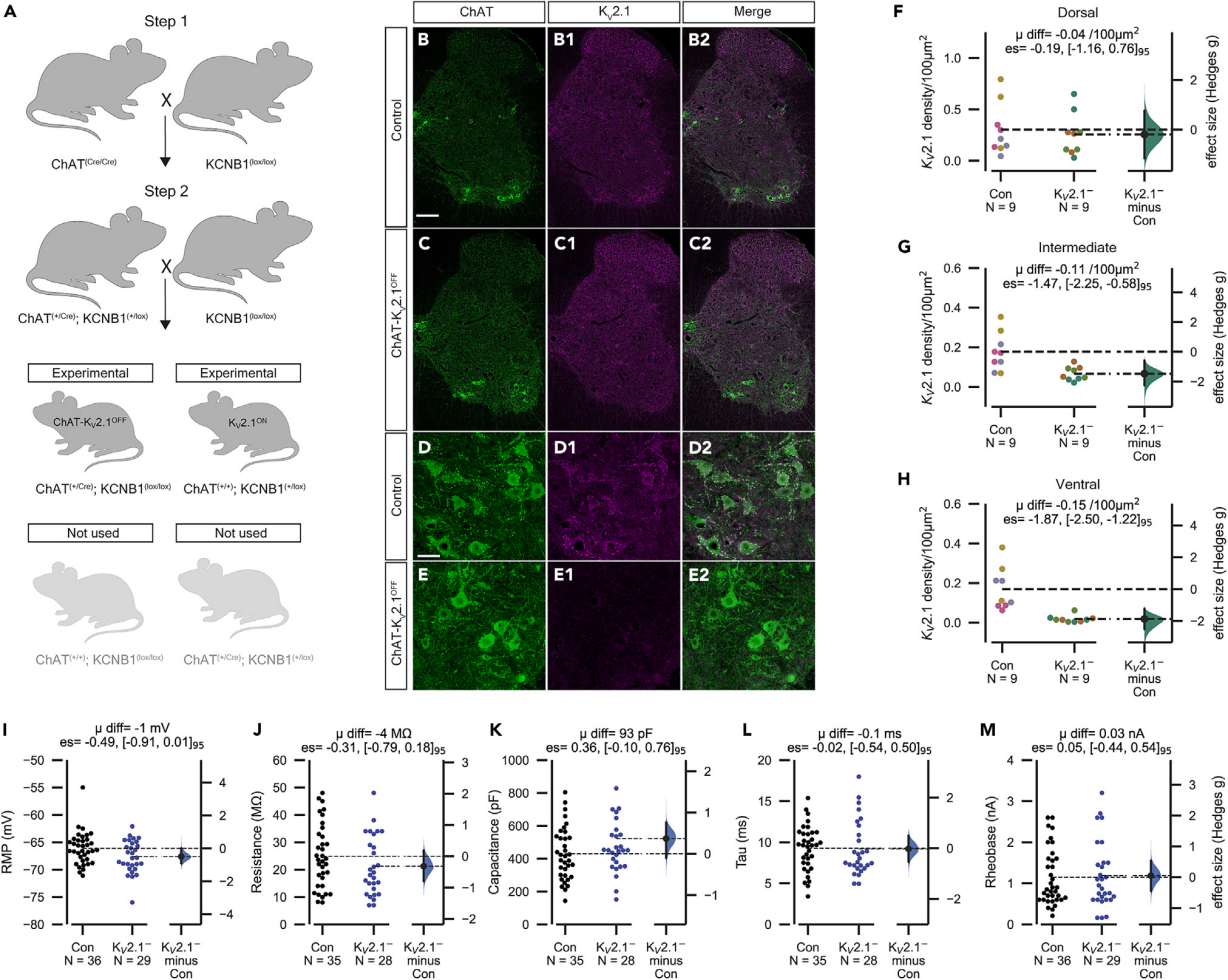
cKO of K_V_2.1 does not alter motoneuron passive membrane properties (A) Two main breeding steps were used to generate experimental animals. In step 1 (first cross), homozygous ChAT^(cre/cre)^ mice were crossed with *KNCB1*^(lox/lox)^ mice to produce heterozygous ChAT^(+/cre)^; *KNCB1*^(+/lox)^ offspring. For step 2, ChAT^(+/cre)^; *KNCB1*^(+/lox)^ mice were bred with *KNCB1*^(lox/lox)^ mice to produce experimental (blue) ChAT^(+/cre)^; *KNCB1*^(lox/lox)^ mice and control mice (gray). (B–C2) Confocal photomicrographs (20× tile scans, 1μm optical section) of spinal cord hemisections from a control (A–C) and ChAT-K_V_2.1^**OFF**^ mouse showing ChAT and K_V_2.1 labeling. (D–D2) 20× z stack images from motor pool of a control mouse spinal cord. (E–E2) As in D-D2, but from a ChAT-K_V_2.1^**OFF**^ mouse. Note the absence of K_V_2.1^+^ puncta on motoneurons following conditional knockout. (F–H) Gardner-Altman estimation plots showing K_V_2.1 density in dorsal (F), intermediate (G) and ventral (H) regions. Density was calculated by counting the number of K_V_2.1 punctae within a region of interest (ROI). The dimensions of the ROI were the same for each section. Each point represents one section, with different fill colors representing sections from different animals. Scale bar, 100 μm in B–C2 and 40 μm in D––E2. Three random, free floating slices (30 μm thickness) were quantified per animal (Control, *N* = 9 slices from 3 animals; ChAT-K_V_2.1^**OFF**^, *N* = 9 slices from 3 animals, all females). (I–M) Gardner-Altman plots comparing resting membrane potential (RMP, I), input resistance (J), whole cell capacitance (K), time constant (tau, L), and rheobase (M) between control and ChAT-K_V_2.1^**OFF**^ mice. Experimental unit (N) = motoneurons recorded from 23 control (*n* = 9 females, 14 males) and 14 ChAT-K_V_2.1^**OFF**^ mice (*n* = 7 females, 7 males).

In order to investigate the contribution of K_V_2.1 channels to motoneuron physiology, we aimed to eliminate K_V_2.1 from motoneurons through multi-generational crossing of ChAT-IRES-Cre (ChAT^(Cre/Cre)^) mice with homozygous floxed *Kcnb1* mice (*Kcnb1*^(f/f)^) to generate ChAT^(Cre/wt)^;*Kcnb1*^(−/−)^, called here ChAT-K_V_2.1^OFF^ mice (Figure 1A). To validate this strategy's efficacy, we proceeded with immunohistochemical labeling using antibodies against ChAT and K_V_2.1 in ChAT-K_V_2.1^OFF^ and littermate controls (ChAT^(w/w)^;*Kcnb1*^(f/f)^ or K_V_2.1^ON^ mice, Figures 1B–1E2). Our analyses showed similar K_V_2.1 punctae density in the dorsal horn of both genotypes (Figure 1F) but large reductions in the intermediate (Figure 1G) and ventral horns (Figure 1H), indicating the strategy was successful, given that cholinergic neurons, including motoneurons are found most densely in these regions. We found no K_V_2.1 labeling on motoneuron somata of ChAT-K_V_2.1^OFF^ mice, suggesting that any residual punctae in the ventral horns were associated with non-cholinergic neurons (Figures 1D–1E2).

Having confirmed that ChAT-K_V_2.1^OFF^ motoneurons are devoid of K_V_2.1, we next performed whole-cell current clamp experiments to define whether the electrical properties of mature (P13-21) ɑ-motoneurons were affected by channel absence. We first assessed the passive membrane properties of large control and ChAT-K_V_2.1^OFF^ motoneurons under resting conditions (no C-bouton activation) and found a small mean difference in resting membrane potential, but input resistance, whole-cell capacitance, time constant, and rheobase were all similar (Figure 1I–1M).

### Motoneuron firing and action potential characteristics are similar in control and ChAT-K_V_2.1^OFF^ mice

**Figure 2:**
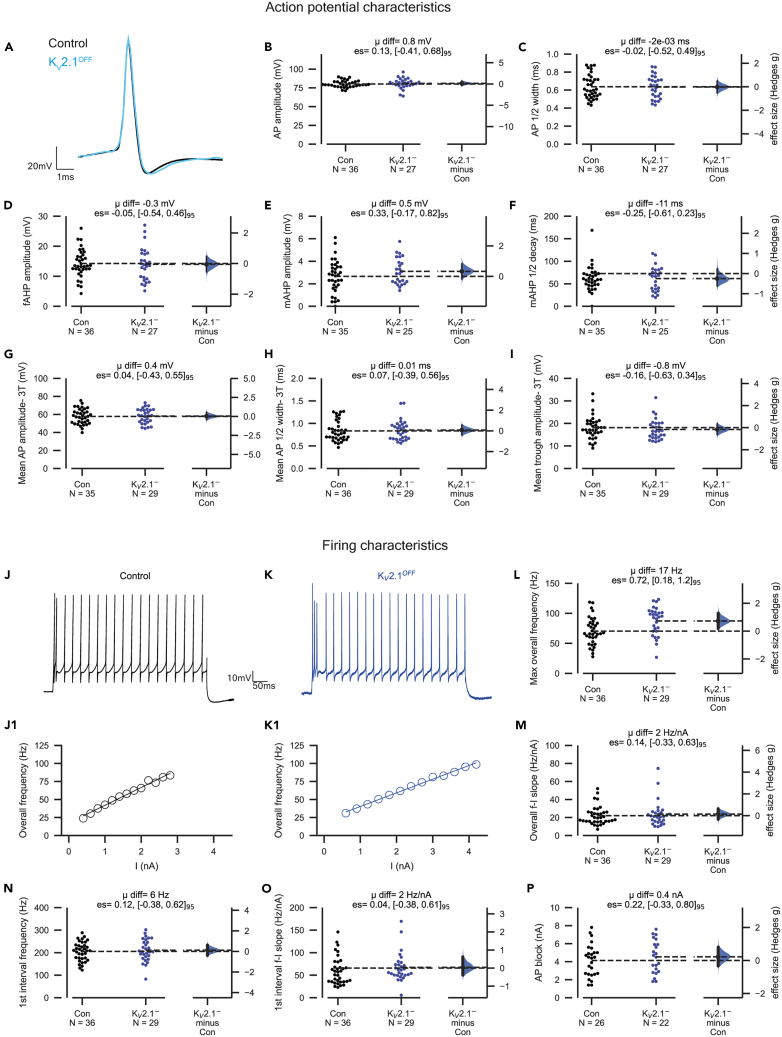
Motoneuron firing and action potential characteristics are similar in control and ChAT-K_V_2.1^OFF^ mice Patch clamp electrophysiology was used to assess various firing and action potential characteristics of lumbar motoneurons in control and ChAT-K_V_2.1^**OFF**^ mice. (A) Representative averages of 15–30 single action potentials (AP) evoked with a 20 ms current pulse in control (Con, black) and ChAT-K_V_2.1^**OFF**^ (blue) motoneurons. (B–F) Gardner-Altman estimation plots for action potential amplitude (B), ½ width (C), fast afterhyperpolarisation amplitude (fAHP, D), medium afterhyperpolarisation amplitude (mAHP, E), mAHP ½ decay time (F). (G–I) As is H–L, but mean of all APs in a 500 ms pulse are taken at 3x threshold (3T) for repetitive firing. AP amplitude is shown in (M), mean AP ½ width-3T (N), and mean AHP-3T (O). Experimental unit (*N*) = motoneurons recorded from 23 control (*n* = 9 females, 14 males) and 14 ChAT-K_V_2.1^**OFF**^ mice (*n* = 7 females, 7 males). Mean difference is abbreviated to “μ diff”, and hedges g estimation statistic is represented by “es”. (J and K) Representative traces from a control (Con, black) and ChAT-K_V_2.1^**OFF**^ motoneuron (blue) at 3x threshold for repetitive firing. (J1–K1) Representative ƒ-I plots for control (black) and ChAT-K_V_2.1^**OFF**^ motoneurons (blue). (L) Gardner-Altman estimation plot for maximum overall frequency, calculated as the mean instantaneous frequency between all spikes within a 500 ms train. (M) The slope of the ƒ-I curve for the overall frequency, calculated from the first linear portion of the plot, as indicated by lines fitted in J1-K1. (N) The maximum instantaneous frequency of the first inter-spike interval in a 500 ms train. (O) As in M, but the ƒ-I curve is only calculated for the first inter-spike interval. (P) The current threshold for depolarizing block (spike failure). Control and ChAT-K_V_2.1^**OFF**^ groups are plotted on the left axes and the bootstrapped sampling distribution (5000 reshuffles) for Hedges g effect sizes are plotted on the right. Each point represents a single motoneuron.

K_V_2.1 delayed rectifier currents are important for maintaining spike shape in many neurons, with numerous studies showing that their block increases spike width and reduces amplitude of both the fast AHP (fAHP) and the spike itself.^20^^,^^34^^,^^35^ However, we found that ChAT-K_V_2.1^OFF^ and control motoneurons had similar spike amplitudes (Figures 2A and 2B), AP ½ widths (Figure 2C), and fAHP amplitudes (Figure 2D).

Although small conductance calcium activated potassium (SK) currents are the main conductances contributing to the medium AHP (mAHP), K_V_2.1 delayed rectifier currents have recently been suggested as an auxiliary mAHP conductance in motoneurons.^22^ Our analyses showed no consistent difference in mAHP amplitudes (Figure 2E) or ½ decay (Figure 2F).

Unlike single evoked spikes, action potentials during repetitive firing are subject to conductances that have longer time constants,^36^ and therefore their morphology may be differentially affected.^21^ Thus, we also assessed mean AP characteristics at 3 times the threshold for repetitive firing, and found that spike amplitude (mean AP amplitude-3T, Figure 2G), AP ½ width (mean AP ½ width-3T, Figure 2H), and inter spike trough (mean trough amplitude-3T, Figure 2I) were similar in control and ChAT-K_V_2.1^OFF^ motoneurons.

Next, we compared repetitive firing properties of motoneurons from both groups. The maximum instantaneous frequency of repetitive firing during the entire 500 ms pulse (max overall frequency, Figures 2J–2L) was higher in ChAT-K_V_2.1^OFF^ motoneurons compared to control, however, the slope of the ƒ-I curve was similar between groups (max overall frequency slope**,** Figure 2M). In addition, the maximum instantaneous frequencies of first two spikes (max 1^st^ interval frequency, Figure 2N), and ƒ-I slopes of this interval (1^st^ interval slope Figure 2O) of control and ChAT-K_V_2.1^OFF^ motoneurons were similar. Taken together, these results suggest that while there is no difference in excitability of the two populations of motoneurons, K_V_2.1 conductances may limit maximum sustained firing rates.

In some neurons, K_V_2 delayed rectifier currents ensure Na^+^ channel recovery by maintaining fast action potential (AP) repolarization kinetics, thereby preventing depolarizing block.^34^^,^^35^ We therefore assessed whether cKO of K_V_2.1 affected the current threshold at which membrane depolarization blocked spike production (Figure 2P). Despite a lack of K_V_2.1, ChAT-K_V_2.1^OFF^ motoneurons entered depolarizing block at similar current thresholds as control motoneurons, suggesting K_V_2.1 contributes little to preventing depolarizing block in spinal motoneurons.

Together, comparisons of firing characteristics and AP morphology from mature (P13-21) control and ChAT-K_V_2.1^OFF^ motoneurons indicate that either K_V_2.1 channels have minimal role in regulating firing capabilities or that there were effective compensatory mechanisms following their loss.

### K_V_2 block by GxTX-1E has minimal effect on motoneuron firing or action potential characteristics

Given that various mechanisms could mask associated deficits in ChAT-K_V_2.1^OFF^ motoneurons, we next assessed the role of K_V_2.1 channels in shaping motoneuron firing characteristics pharmacologically. We used the Chinese tarantula toxin guangxitoxin-1E (GxTX-1E), which at 100 nM potently and selectively inhibits K_V_2 channels.^20^^,^^22^^,^^34^^,^^37^^,^^38^ Because K_V_2.1 channels have recently been suggested to play a significant role in regulating motoneuron firing, we hypothesized GxTX-1E would significantly alter firing characteristics in control but not ChAT-K_V_2.1^OFF^ motoneurons.

To confirm that in our hands GxTX-1E does not have significant effects on passive membrane properties of wild-type motoneurons,^20^^,^^22^ we first measured these effects in both our control and ChAT-K_V_2.1^OFF^ motoneurons by measuring voltage responses to sub-threshold and threshold current steps (Figures S1A–S1E). In line with previous work, there were no effects of the toxin on resting membrane potential in either condition (Figure S1B). Similarly, we found no consistent effect of GxTX-1E on input resistance (Figure S1C) or whole-cell capacitance (Figure S1D) in either control or ChAT-K_V_2.1^OFF^ motoneurons. In contrast, we saw small increases in the rheobase of both ChAT-K_V_2.1^OFF^ and control motoneurons (Figure S1E), suggesting that GxTX-1E had effects on conductances other than K_V_2.1.

**Figure 3:**
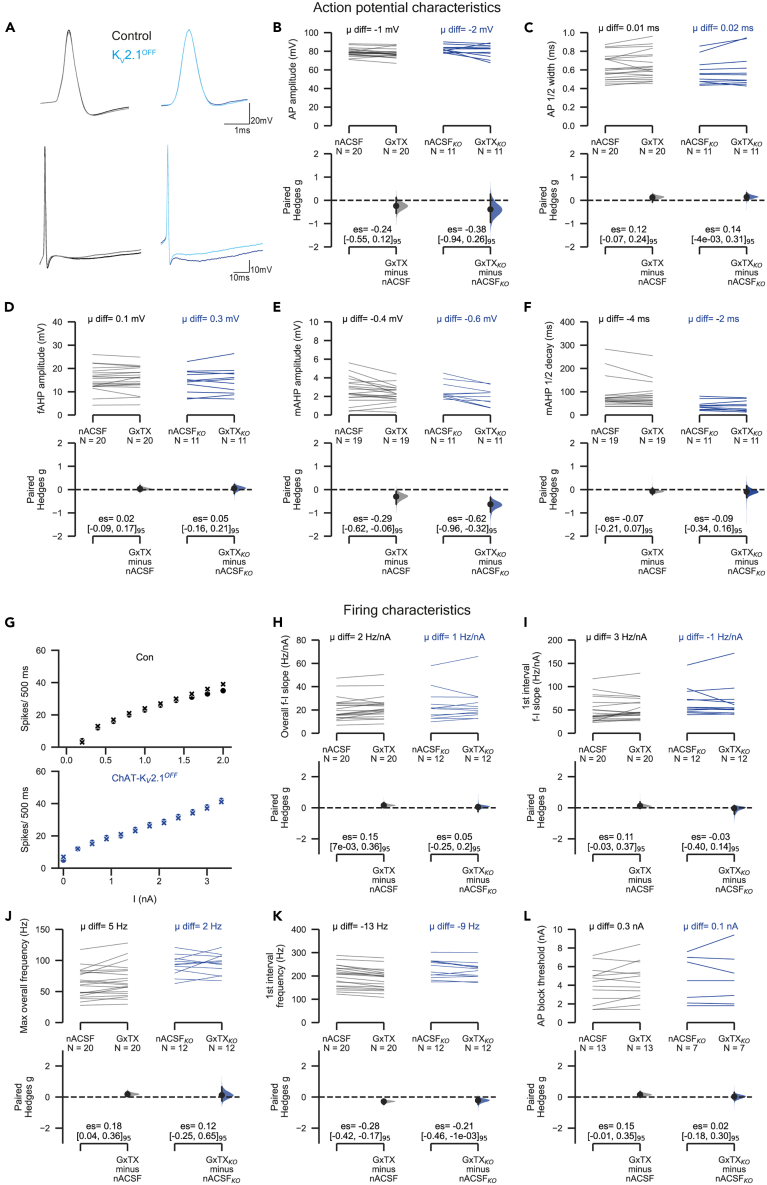
K_V_2 block by GxTX-1E has minimal effect on motoneuron maximal firing or action potential characteristics See also Figures S1–S3. (A–F) Comparing the effects of K_V_2 inhibition with GxTX-1E on action potential characteristics. (A) The upper panel shows representative single evoked AP traces from control (left) and ChAT-K_V_2.1^**OFF**^ motoneurons (right). The dark colors (black, blue) show spike morphology in the presence of nACSF only and the light colors (gray, sky blue) show morphology after 10 min perfusion with 100 nM GxTX-1E. The lower panels show longer sweeps in order to visualize the mAHP. (B-F) Paired Hedges g for control (left) and ChAT-K_V_2.1^OFF^ (right) motoneurons: (B) shows the spike amplitude, (C) is action potential ½ width, (D) the fAHP amplitude, (E) is the mAHP amplitude, and (F) is the mAHP ½ decay time. (G) Scatterplots of spike number in response to increasing current input for representative motoneurons from control (upper graph, black) and ChAT-K_V_2.1^**OFF**^ mice (lower graph, blue). Spike number in motoneurons perfused with nACSF only is plotted using “O” and following 10 min GxTX-1E is plotted with “X”. (H–L) Paired Hedges g for overall f-I slope (H), 1^st^ interval f-I slope (I), maximum overall frequency (J), maximum 1^st^ interval frequency (K), and AP block threshold (L) in control (left) and ChAT-K_V_2.1^OFF^ (right) motoneurons are shown in Cumming paired estimation plots with Hedges g distributions shown in the following text. Number of animals used was as follows: control = 13 (6 females, 7 males), ChAT-K_V_2.1^**OFF**^**=** 6 (4 females, 2 males). Experimental unit (*N*) = motoneurons. Mean difference is abbreviated to “μ diff”, and hedges g estimation statistic is represented by “es”. See also Figures S1 and S2.

We next measured the effects of GxTX-1E on action potential morphology. We found no significant effects on AP amplitude (Figures 3A and 3B), AP half-width (Figure 3C), or fAHP amplitude (Figure 3D). Recent evidence suggested that K_V_2.1 conductances contribute to the mAHP.^22^ In agreement, we found that GxTX-1E had a medium effect (Hedge’s g = 0.6) on mAHP amplitudes in ChAT-K_V_2.1^OFF^ motoneurons, and a small effect (Hedge’s g = 0.3) in control motoneurons (Figure 3E). There were no effects of the toxin on mAHP half decay (Figure 3F). Because GxTX-1E-mediated decreases in mAHP amplitude were seen in both control and ChAT-K_V_2.1^OFF^ motoneurons, it is likely that GxTX-1E acted on conductances other than K_V_2.1.

GxTX-1E block of K_V_2 channels also had little effect on motoneuron repetitive firing properties. The slopes of the ƒ-I relationship for overall and initial frequency were unaltered by GxTX-1E in both ChAT-K_V_2.1^OFF^ (Figures 3G–3I) and control motoneurons. Similarly, we found little effect of GxTX-1E on the maximum overall firing frequency for either ChAT-K_V_2.1^OFF^ or control motoneurons (Figure 3J). However, GxTX-1E did lead to small reductions in the maximum first interval firing frequency in both ChAT-K_V_2.1^OFF^ and control motoneurons (Figure 3K), again suggesting that the toxin was acting on conductances other than K_V_2.1.

In cells in which K_V_2.1 has a significant conducting role, GxTX-1E reduces the threshold for depolarizing block of action potentials.^34^^,^^35^^,^^39^ However, GxTX-1E had no effect on the threshold for AP block in either ChAT-K_V_2.1^OFF^ or control motoneurons (Figure 3L).

In summary, apart from small increases in rheobase and decreases in frequency of the first interspike interval and mAHP amplitude in both ChAT-K_V_2.1^OFF^ and control motoneurons, GxTX-1E had little effect on firing capabilities or AP characteristics. Importantly, GxTX-1E effects were seen in both genotypes, suggesting that the toxin was acting on conductances other than K_V_2.1 (possibly K_V_2.2, see in the following text).

### K_V_2 block increases excitability of cortex layer V pyramidal neurons

The results to this point suggest that under resting conditions (no C-bouton activation), K_V_2.1 does not play a significant role in regulating motoneuron firing in motor mature (P13-21) mice. Because these results were not consistent with studies in younger animals,^20^^,^^21^^,^^22^ we next proceeded with a positive control by testing our protocol on layer V cortical pyramidal neurons, in which K_V_2.1 channels have significant electrophysiological functions.^38^^,^^39^^,^^40^

We found that GxTX-1E caused significant changes to individual pyramidal neuron action potential characteristics with large effect sizes in pyramidal neurons (Figure S2). As demonstrated in similar studies assessing the effect of inhibiting K_V_2.1 in cortical and other brain neurons,^35^^,^^38^^,^^39^^,^^40^^,^^41^ we found large increases in AP ½-width (Figure S2C), as well as decreases in spike amplitude (Figures S2A and S2B) and fAHP (Figure S2D). GxTX-1E also caused a small decrease in mAHP amplitude (Figure S2E), but mAHP ½-decay was not affected (Figure S2F).

GxTX-1E also had effects on repetitive firing of cortical neurons, leading to increases in overall ƒ-I slope (Figures S2G–S2I) and initial interval ƒ-I slope (Figure S2J). There was no consistent effect on maximum firing frequencies: both the overall (Figure S2K) and initial (Figure S2L) maximum frequencies were similar in both conditions. This resulted from the neurons reaching depolarizing block at significantly lower current thresholds (Figure S2M).

In summary, we found that GxTX-1E block of K_V_2 channels had little effect on the firing capacity of mature control and ChAT-K_V_2.1^OFF^ motoneurons. But the same experiments in layer 5 cortical pyramidal neurons greatly increased excitability and maximum firing, in line with previous studies. The clear differences in paired sample effect sizes (Hedges g) on the parameters assessed suggest that, compared to pyramidal neurons, K_V_2 channels (both 2.1 and 2.2) play only a minor role in regulating motoneuron repetitive firing (Figure S2N).

### Post-natal clustering does not significantly alter K_V_2 regulation of motoneuron firing

During post-natal motoneuron development, K_V_2.1 channels organize into high-density macroclusters,^8^ a configuration associated with non-conducting channels^24^ and that may play a role in neurodevelopment in the forebrain.^42^ Because our patch clamp experiments were done at an age (post-natal week 3) at which K_V_2.1 clusters appear mature,^8^ we investigated whether developmental clustering of channels might contribute to differences in our results compared to studies in young motoneurons.^20^^,^^21^^,^^22^

**Figure 4:**
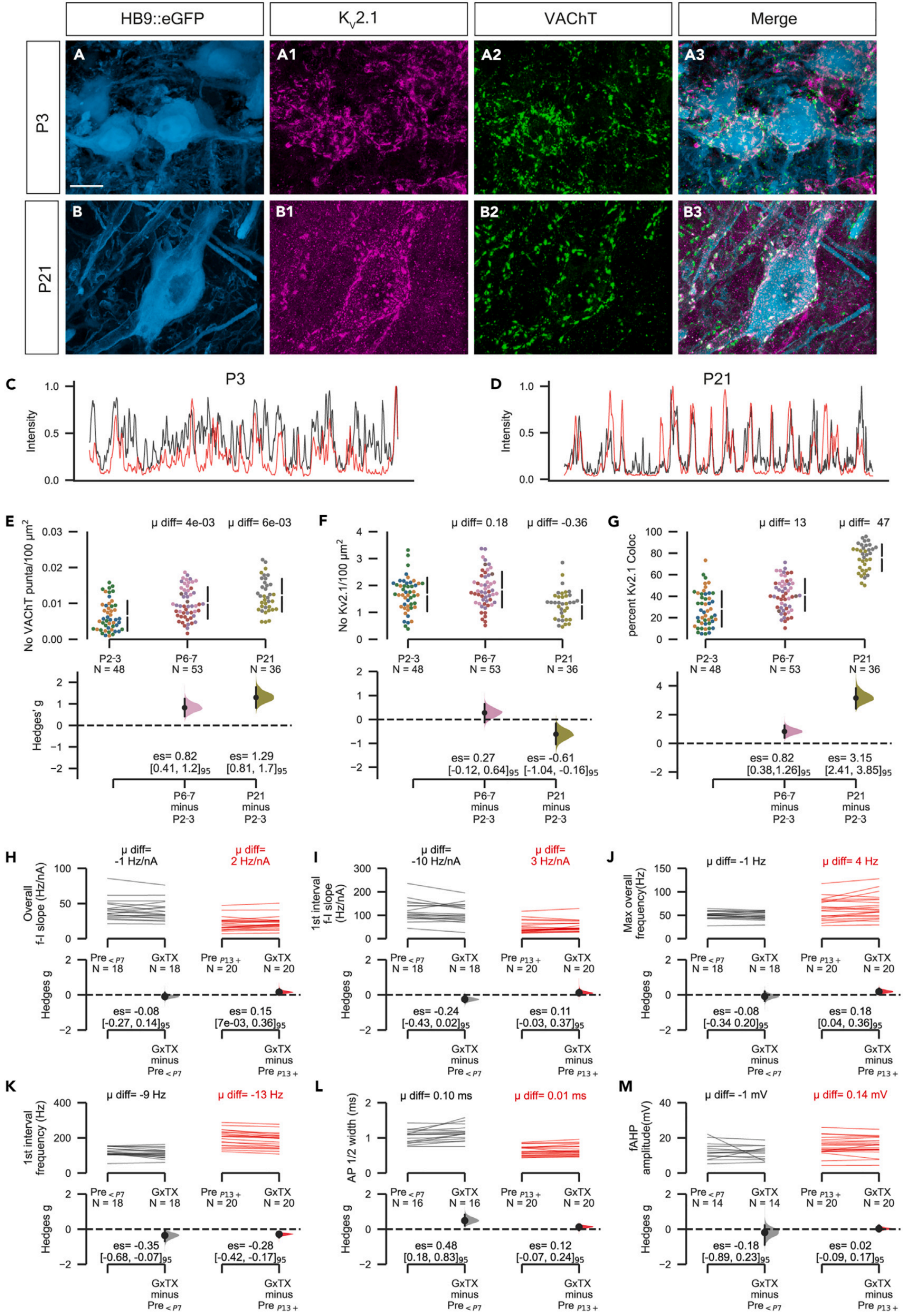
Post-natal clustering does not significantly alter K_V_2 regulation of motoneuron firing (A and B) 3D projections of Z-stacks taken at 60x on a confocal microscope showing raw GFP expression under the Hb9 promotor (A and B), immuno-labeling of K_V_2.1 (A1 and B1) and VAChT (A2 andB2, depicting presynaptic C-boutons**)**. Images merged in (A3 and B3). (C and D) Intensity plots (intensity/maximum intensity) showing the distribution of VAChT and K_V_2.1 signal around the perimeter of a 60× confocal image (1μm section) of a representative motoneuron from a P3 (F) and P21 (G) mouse. (E–G) Gardner-Altman estimation plots for the density of VAChT^+^ C-boutons (E), density of K_V_2.1 (F), and % K_V_2.1 clustered to C-boutons on individual motoneurons from throughout development (G). Values for individual cells are plotted (color coded by animal) on the upper axes and the hedges g effect sizes for P6-7 vs. P2-3 and P21vs P6-7 on the lower axes. Note that measurements were taken at P2, 3, 6, 7 and P21, however cells were analyzed in 3 age groups. A total of 137 motoneurons were sampled from 8 animals (P2-3: 3 mice, *N* = 48; P6-7: 3 mice *N* = 51; P21: 2 mice, *N* = 36; all females). (H–M) Cumming paired estimation plots showing paired Hedges g for overall frequency slope (H), 1^st^ interval frequency slope (I), maximum overall frequency (J), maximum 1^st^ interval frequency (K), AP ½ width (L), and fAHP (M) in young (P2-7, left) and motor mature (P13-21, right) motoneurons. The number of motoneurons and animals used for each age was as follows: P2-3 = 8 cells from 4 mice (2 males, 2 females); P6-7 = 10 cells from 5 mice (2 males, 3 females; 9 animals total for the <P7 group), for mature motoneurons (P13+) 13 were mice were used (6 females, 7 males). Scale bar in (A), 20μm. Mean difference is abbreviated to “μ diff”, and hedges g estimation statistic is represented by “es”. See also Figure S3.

We first used immunohistochemistry to quantitatively assess the expression and membrane localization of K_V_2.1 channels in lumbar motoneurons from mice aged P2-P3 (neonatal, Figures 4A–4A3), P6-P7 (transition) and P21 (motor mature, Figures 4B–4B3). The density of VAChT^ON^ C-boutons increased with development (Figure 4C), while K_V_2.1 density (# puncta pre 100μm^2^) decreased between P3 and P21 (Figure 4D). We also saw an increase in the percentage of K_V_2.1 localized opposite C-boutons with age (Figure 4E). These data show that in neonatal motoneurons, K_V_2.1 channels are mainly organized in dispersed microclusters (Figure 4F), many of which are not associated with C-boutons (Figure 4G). As post-natal development progresses and motoneurons (Smith and Brownstone, 2020) and motor behaviors mature,^43^ large macroclusters of K_V_2.1 channels form opposite C-bouton synapses.

We next compared the effects of GxTX-1E on immature and mature motoneuron firing and AP characteristics. As with mature motoneurons (described previously), GxTX-1E had minimal effect on overall ƒ-I slope (Figure 4H), first interval ƒ-I slope (Figure 4I), maximum overall frequency (Figure 4J), or maximum first interval frequency (Figure 4K). GxTX-1E increased the AP ½-width by 0.10 ms in young motoneurons compared to 0.01 ms in mature motoneurons (Figure 4L), suggesting a contribution of K_V_2 conductances to action potential repolarization at this stage. As with mature motoneurons, there was no effect of GxTX-1E on fAHP amplitude in young motoneurons (Figure 4M). Together, these results show that K_V_2 conductances have minimal effects even in neonatal motoneuron firing but do have a significant effect on AP repolarization in neonatal motoneurons.

We next attempted to measure K_V_2 currents in voltage clamp experiments with sodium and calcium currents blocked. Being concerned that the large amplitudes of the potassium currents would preclude quantification because of the large voltage error induced by series resistance in the presence of high-amplitude currents, we sought, through a series of 2-electrode voltage clamp experiments, to determine whether it would be reasonable to make even a qualitative assessment (Figure S3A). When current was passed through the V-clamp electrode, while simultaneously measuring voltage with a second, voltage follower, electrode, the voltage error was indeed very high for currents above 2–3 nA (albeit the true voltage error was less than predicted, Gray and Santin, 2023). Since K currents in motoneurons can be as large as 30 nA (Figures S3B and S3C), we could not accurately measure the I-V relationship. However, we reasoned that, although quantification would be inaccurate, if we saw no change in currents with GxTX-1E, then there would be little in the way of K_V_2 conductance. Indeed, while there is potential evidence of a reduction in total potassium currents (“measured” as ∼5%) at early post-natal stages, there was none in the 3^rd^ post-natal week (Figure S3D). These results are consistent with the aforementioned results, showing little effect of K_V_2 conductances on motoneuron firing.

Taken together, even at neonatal ages when there is less clustering, K_V_2 channels contribute minimally to overall potassium currents, and at mature stages, their conductance does not contribute to motoneuron firing. That is, the lack of effects on motoneuron firing of both the developmental cKO of K_V_2.1 channels and acute pharmacological block of K_V_2 channels do not appear to be due to developmental differences such as channel clustering.

### Muscarine-induced increase in excitability is preserved in ChAT-K_V_2.1^OFF^ motoneurons

Motoneuron K_V_2.1 channels are opposed to, and thought to be modulated by C-boutons via M2 muscarinic acetylcholine receptors.^7^^,^^22^ Our data to this point suggest that the conducting role for K_V_2.1 is minimal in the absence of muscarinic activation. To assess whether K_V_2.1 conductances play a role in mediating the effects of C-boutons, we proceeded to compare motoneuron responses to muscarine in control and ChAT-K_V_2.1^OFF^ mice.

In neonatal motoneurons, exogenously applied muscarine has mixed effects due to the different subtypes (M2/M3) of receptors.^44^ In agreement with those findings, we found that P2-P7 motoneurons (*n* = 9) had large depolarizations in response to muscarine (10 μM). However, in motoneurons from P13 and older mice (*n* = 10), muscarine did not affect resting membrane potential (Figure S4). Rather, the effects of muscarine were dominated by those shown to be due to M2 receptor activation^3^ as outlined in the following text.

Perfusion of mature control and ChAT-K_V_2.1^OFF^ motoneurons with 10 μM muscarine had little effect on AP amplitude (Figures S4C and S4D), ½-width (Figure S4E), or fAHP amplitude (Figure S4F). However, the mAHP amplitude (Figure S4G) and ½-decay time (Figure S4H) were significantly decreased in both control and ChAT-K_V_2.1^OFF^ motoneurons.

**Figure 5:**
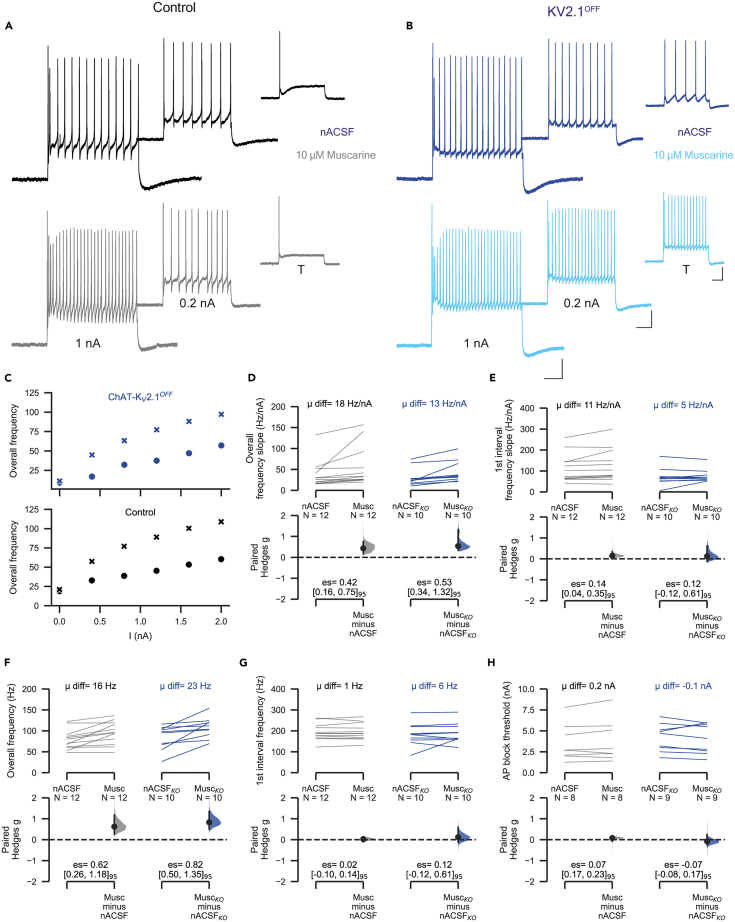
Muscarinic induced increase in excitability is preserved in ChAT-K_V_2.1^OFF^ motoneurons See also Figures S4 and S5. (A and B) The changes in firing characteristics following perfusion with 10μM muscarine were compared for motoneurons of control (A) and ChAT-K_V_2.1^OFF^ mice (B). Representative traces at threshold (T), 0.2 nA and 1nA above threshold before (nACSF, black and dark blue traces) and after perfusion with muscarine (10 μM, gray and sky blue). (C) Scatterplots showing the overall frequency in response to increasing current inputs in control (upper) and ChAT- K_V_2.1^OFF^ (lower) motoneurons (circles control, x muscarine). (D–H) Paired Hedges g for overall frequency slope (D), 1^st^ interval frequency slope (E), maximum overall frequency (F), maximum 1^st^ interval frequency (G), and AP block threshold (H) in control (left) and ChAT-K_V_2.1^OFF^ (right) motoneurons are shown in Cumming paired estimation plots. Experimental unit (N) = motoneurons. Number of animals used was as follows: control = 8 (2 females, 6 males), ChAT-K_V_2.1^**OFF**^ = 6 (2 females, 4 males). Scale bars in A and B: horizontal, 100 ms, vertical, 20 mV. Mean difference is abbreviated to “μ diff”, and hedges g estimation statistic is represented by “es”. See also Figure S4.

In agreement with previous work in control motoneurons,^3^ and in line with the reduction in mAHP conductances, muscarine affected firing of mature motoneurons from both ChAT-K_V_2.1^OFF^ (Figure 5A) and K_V_2.1^ON^ (Figure 5B) mice. Muscarine increased overall ƒ-I slope (Figures 5A–5D), but not the ƒ-I slope of the first interval (Figure 5E), and induced moderate increases in maximum overall firing frequency (Figure 5F). The first spike interval frequency was not altered by muscarine (Figure 5G). That is, the absence of K_V_2.1 channels did not affect motoneuron responsiveness to muscarine.

As K_V_2 channels have been shown in other neurons to prevent depolarizing block to maintain firing during high synaptic drive, we asked whether muscarinic receptor activation might modulate K_V_2.1 to increase the current threshold at which spike output is blocked. However, muscarine had no effect on depolarizing block threshold in either control or cKO motoneurons (Figure 5H).

Taken together, these results show that K_V_2.1 is not required for muscarine-evoked increases in motoneuron excitability.

### Motor behavior and C-bouton amplification is preserved in ChAT-K_V_2.1^OFF^ mice

Despite our *in vitro* data showing K_V_2.1 channels are not necessary for motoneuron amplification (including following muscarine application), we tested whether our negative electrophysiology results translated to behavior, i.e., whether ChAT-K_V_2.1^OFF^ mice would show deficits in high force tasks. Voluntary motor behavior over a chronic period was assessed by individually housing male (Figure S5A) and female (Figure S5B) mice of both genotypes in cages with *ad libitum* access to a running wheel for either 23 days (males) or 32 days (females). Male control and ChAT-K_V_2.1^OFF^ mice ran similar daily distances throughout the 23 day period. We recorded females for a longer duration as their distances (which are much greater than males) seemed to change over the course of 5 weeks. Although at no time point were the female ChAT-K_V_2.1^OFF^ significantly different from control mice, the control mice—unlike their male counterparts—seemed to increase their running distances over time, with the slope over five weeks being significantly greater than that of ChAT-K_V_2.1^OFF^ mice (*p* = 0.003).

We next examined the running capacity of mice on a treadmill inclined 15° to increase locomotor force demands. The maximum speed for male ChAT-K_V_2.1^OFF^ group was on average 13% (6 cm/s) slower than control mice. Although the effect size was large, the 95% confidence interval was wide and crossed 0 (CI_95_ = [-1.84, 0.07]), indicating a relatively low degree of certainty in the magnitude of this effect (Figure S5C). There was no difference in this measure for female mice (Figure S5F).

Other behavioral measures, including the maximum distance run at 60% of maximum speed (Figures S5D and S5G) and grip strength (Figures S5E and S5H), were similar in both genotypes for male (Figures S5D and S5E) and female (Figures S5G and S5H) mice.

In short, we were unable to detect any significant deficits in either male or female ChAT-K_V_2.1^OFF^ mice using these particular tasks.

**Figure 6:**
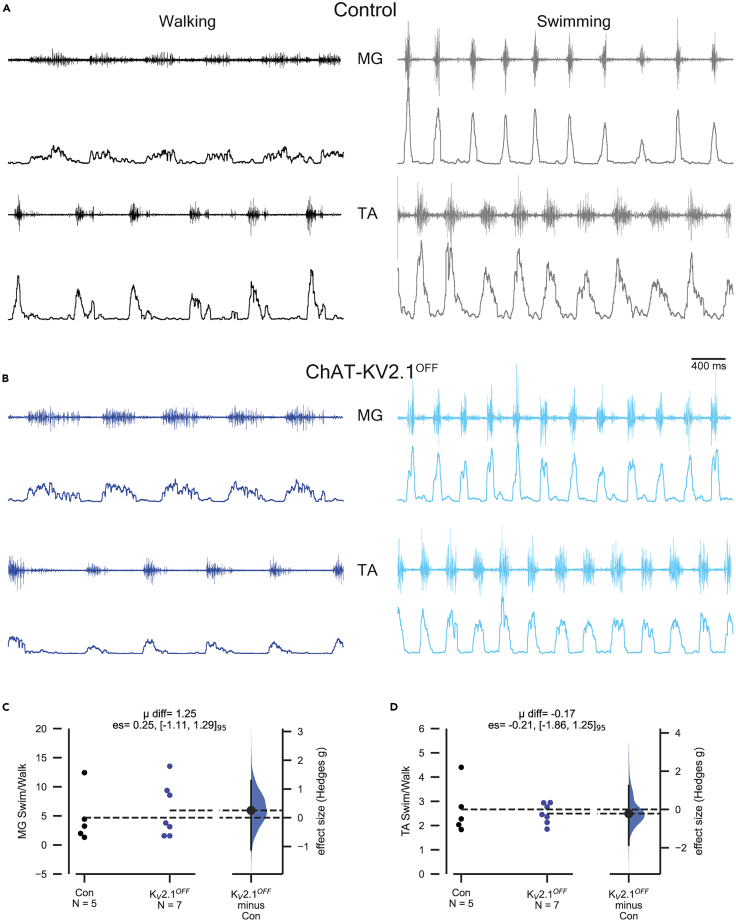
Motor behavior and C-bouton amplification is preserved in ChAT-K_V_2.1^OFF^ mice See also Figure S5. (A and B) Representative medial gastrocnemius (MG) and tibialis anterior (TA) EMG traces from a control (A) and ChAT-K_V_2.1^OFF^ mouse (B). For control mice (A), black traces are those recorded during walking at 0.15 m/s and gray traces are during swimming. In ChAT-K_V_2.1^OFF^ mice (B), blue traces are walking at 0.15 m/s and sky blue is swimming. For each muscle, top traces are raw EMGs and lower traces are corresponding RMS amplitudes. (C and D) Gardner-Altman estimation plots for MG and TA. Experimental unit (*N*) = animals, all were females. Mean difference is abbreviated to “μ diff”, and hedges g estimation statistic is represented by “es”. See also Figure S5.

C-bouton mediated amplification can be specifically assessed *in vivo* by comparing EMG amplitudes during walking and swimming: when C-bouton synaptic transmission is genetically perturbed, the ratio of the EMG amplitude during swimming vs. walking is reduced compared to mice with functional C-bouton signaling.^4^^,^^45^^,^^46^ Thus, if K_V_2.1 were critical to C-bouton function, a similar decrease in motor amplification would be expected following K_V_2.1 cKO in motoneurons. However, we found no difference in EMG amplification during swimming between control and ChAT-K_V_2.1^OFF^ female mice in either the extensor medial gastrocnemius (MG) muscle (Figures 6A–6A2) or the flexor tibialis anterior (TA) muscle (Figures 6B–6B2, male mice were not studied).

In summary, the only behavioral effect of eliminating K_V_2.1 from cholinergic neurons that we observed was that of a possible training effect in female mice given *ad libitum* access to a running wheel. Notably, these data suggest that either K_V_2.1 channels are not necessary for motoneuron amplification—the key reported role of C-boutons—or that their absence can be compensated to maintain behavioral homeostasis.

### K_V_2.2 is expressed in the spinal cord and co-localizes with K_V_2.1 opposite to C-boutons

**Figure 7:**
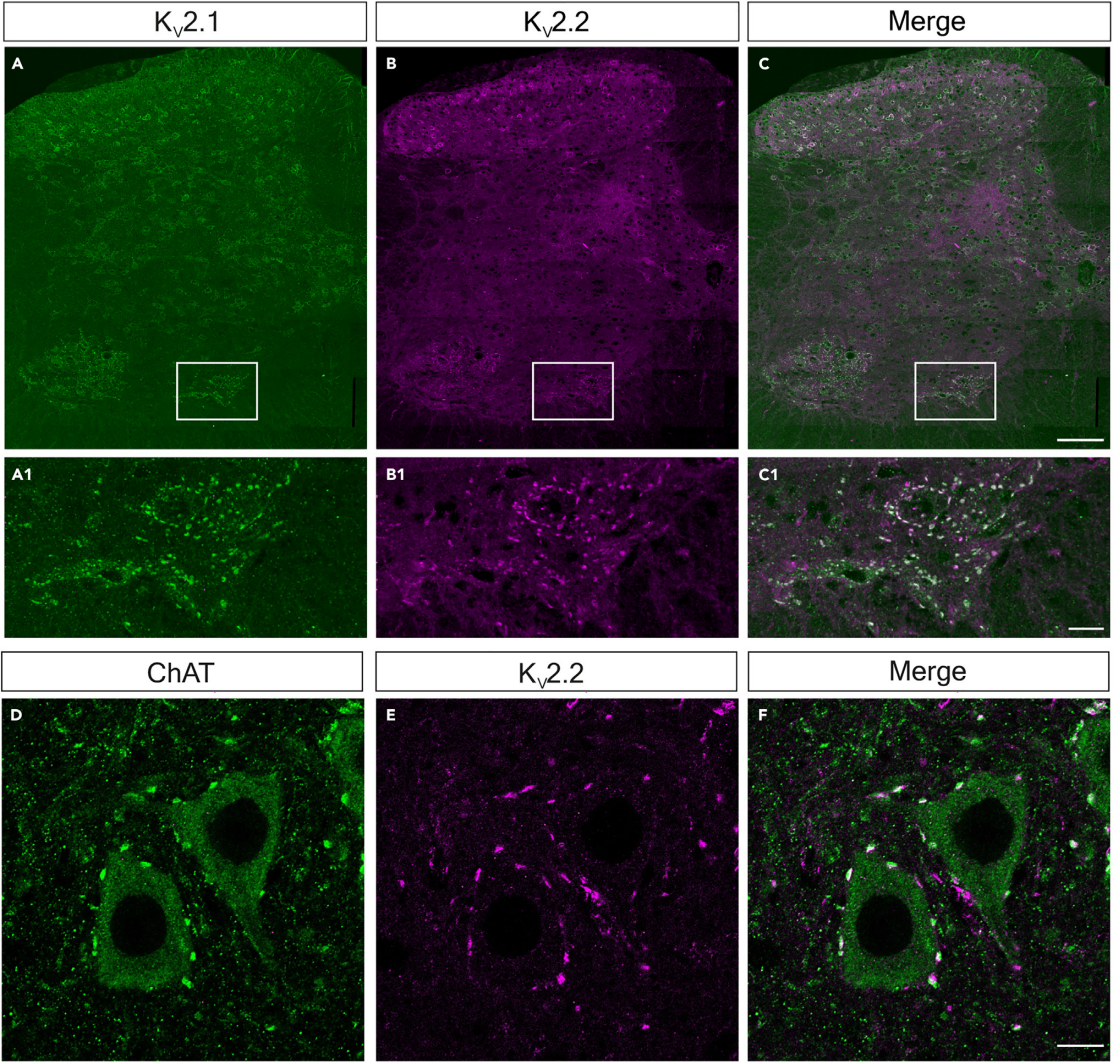
K_V_2.2 is expressed in the spinal cord and co-localizes with K_V_2.1 opposite to C-boutons See also Figure S6. (A–C) 40× confocal tiled, z stack projection images (5 × 1μm slices) of the lumbar spinal cord stained for K_V_2.1 (A) and K_V_2.2 (B); merged image shown in (C). (A1–C1) Boxes depicted in A–C, expanded. (D–F) 40× confocal single optical sections showing K_V_2.2 (D) expression opposite VAChT^+^ C boutons (E), with a merge of both channels in (F). Scale bars in (A–C), 80 μm, (A1–C1), 25 μm, (D–F), 10 μm. See also Figure S7.

The lack of behavioral deficits, particularly in motoneuron amplification following cKO of K_V_2.1 were surprising findings considering that these channels are prominent at all C-bouton synapses and thought to be an integral part of the synaptic machinery.^21^^,^^22^ Although K_V_2.1 is considered to be the predominant K_V_2 subunit in motoneurons, in many brain neurons K_V_2.1 subunits share some functional homology and are co-expressed with K_V_2.2 subunits.^29^^,^^35^^,^^39^^,^^40^^,^^41^ We therefore proceeded with immunohistochemistry experiments to identify whether K_V_2.2 is also expressed in motoneurons. We found that K_V_2.2 puncta were abundant in the spinal cord and often but not always co-localized with K_V_2.1 (Figures S6A–S6F). In the dorsal horn (Figures S6A–S6C1), K_V_2.1 was predominantly expressed in the deeper laminae, whereas K_V_2.2 positive cells were concentrated in the superficial laminae. In the deep dorsal/intermediate laminae (Figures S6D–S6F1), K_V_2.2 expression was mostly confined to medial regions, and as well as forming large clusters on somata, an area of smaller, more diffuse puncta was located lateral to the dorsal columns (Figures S6D–S6F1). In the same region, K_V_2.1 puncta were distributed across the medio-lateral axis, mainly in neurons without K_V_2.2 labeling. In the ventral horn (Figures 7A–7F), K_V_2.2 seemed to be exclusively confined to motoneurons, where labeling was co-localized with K_V_2.1. Co-labeling experiments with ChAT confirmed that indeed K_V_2.2 clusters are opposed to C-boutons (Figures 7D–7F). Thus, K_V_2.2 channels are clearly co-localized with K_V_2.1 channels at motoneuron membranes in apposition to pre-synaptic C-boutons.

## Discussion

K_V_2.1 channels are expressed by spinal motoneurons where they largely aggregate opposite C-bouton synapses,^7^ clustering in the post-natal period as the motor system matures.^8^^,^^43^^,^^47^^,^^48^ K_V_2.1 delayed rectifier currents have been implicated as crucial regulators of motoneuron firing and C-bouton amplification of motor output. Here, we show that K_V_2.1 does not regulate firing in mature motoneurons. Furthermore, in the absence of K_V_2.1 channels in motoneurons, C-bouton amplification of motor output during behavior is preserved. We also show that motoneurons co-express K_V_2.2 and suggest that K_V_2 channels function as non-conducting proteins in mature motoneurons. Thus, our data challenge current concepts of motoneuron physiology and raise new questions about the role of these prominent channels in motoneuron behavior.

### Co-expression of K_V_2.1 and K_V_2.2 in motoneurons

The structure and function of neuronal K_V_2 channels has been studied for over 3 decades, since K_V_2.1 was shown to be expressed in rat brain.^12^ K_V_2.2 channels were discovered shortly afterward, and, although the two subtypes have similar electrophysiological properties, substantial differences in their expression profiles across the brain were initially reported.^13^ In addition to their expression being in different neurons, K_V_2.2 (initially called “CDRK”) expression seemed to be restricted to neuronal processes rather than somata.^14^ The prominence of K_V_2.1 expression led these channels to become the primary focus of further studies of K_V_2 regulation of neuronal excitability.

However, this characterization of K_V_2.2 was based on an unfortunate cloning error, and it was later demonstrated that both K_V_2 isoforms are “co-localized in the somata and proximal dendrites of cortical pyramidal neurons and are capable of forming heteromeric channels”.^49^ Nonetheless, much of the focus has remained on K_V_2.1 and only recently has K_V_2.2 received increasing attention.^29^^,^^30^^,^^39^^,^^40^^,^^49^ But K_V_2.2 has remained largely unexplored in motoneurons (see Stewart et al.).^50^ Here, we show that K_V_2.2 is also expressed post-synaptically to C-boutons, is co-clustered with K_V_2.1, and as such, should be taken into consideration when investigating C-bouton function. Whether K_V_2.2 expression increases in ChAT-K_V_2.1^OFF^ motoneurons as a compensatory mechanism remains unknown.

### Reconciling differences with studies of K_V_2.1 function in immature motoneurons

Our experiments were initially performed with the assumption that K_V_2.1 was the predominant K_V_2 subtype in motoneurons and that it had a significant conducting role. We were therefore surprised to find that the only difference in mature ChAT-K_V_2.1^OFF^ motoneurons compared to controls was a capacity to fire at higher rates. The reason for these higher firing rates is not clear, but they may be due to disruption of the post-synaptic site (see in the following text). But given the filtering properties of muscle fibers, these high-sustained frequencies may not be relevant to muscle contraction (Enoka and Farina, 2021).

If K_V_2 channels were to have an electrical role, acute inhibition with GxTX-1E would significantly alter firing characteristics of both control (K_V_2.1^ON^/K_V_2.2^ON^) and ChAT-K_V_2.1^OFF^ motoneurons (ChAT-K_V_2.1^OFF^/K_V_2.2^ON^), as it did in wild-type pyramidal neurons. However, the only consistent effect we found in response to toxin was a relatively small (less than 10% in all neurons) reduction in the instantaneous frequency of the initial two spikes in a train. We saw the same effect in ChAT-K_V_2.1^OFF^ motoneurons, suggesting K_V_2.2 currents also contribute to maintaining high initial firing frequencies. Functionally, this could be significant as small increases in initial firing frequencies are known to significantly increase the rate of muscle force production.

We note that these findings contrast with those indicating that K_V_2 inhibition alters motoneuron firing and excitability.^20^^,^^21^^,^^22^ Fletcher et al. (2017) used GxTX-1E to block motoneuron K_V_2 channels in P4 mice, suggesting that K_V_2 conductances maintain narrow spikes and repetitive firing at low current inputs and increase firing frequencies. Romer et al. (2019) assessed the contribution of K_V_2 to regulation of motoneuron firing in spinal cord slices from P8-12 rats, which are approaching motor maturity. They reported that blocking K_V_2 channels with stromatoxin led to unsustainability of repetitive firing, a reduction in excitability (*f-*I slope), and a slowing of the rising and falling phases of action potentials (simlar to Fletcher et al., 2017). And Nascimento et al. (2020) showed in young (P2-P7) motoneurons that chemogenetic activation of V0_C_ interneurons (the neuronal source of C-boutons) decreases spike width, while increasing maximal firing rates, the current required for depolarizing block, and mAHP; these effects were all blocked by GxTX-1E. They thus suggested that clustered K_V_2.1 channels underlie these M2-mediated electrophysiological effects and are recruited by C-boutons for motor amplification.

Our results in young motoneurons are in keeping with the aforementionned studies. For example, we too showed that K_V_2 channels contribute to spike width in early post-natal life, during a period when K_V_2.1 channels and motor control are immature.^8^^,^^43^ But this contribution is minimal in mature motoneurons. And the one study aforementioned in an intermediate developmental stage (Romer et al., 2019) used stromatoxin, which blocks K_V_2 channels but also has off target effects (K_V_4.2 channels with a low IC_50_) that make interpretation of current clamp data challenging.^34^^,^^51^

Another difference between our study and that of Nascimento et al. (2020) is the specificity of C-bouton activation. While their chemogenetic activation would limit off target effects, we used muscarine—an agonist of all muscarinic receptors that has been shown to have mixed effects on early post-natal motoneurons.^3^^,^^44^ Indeed, we found that in young motoneurons, muscarine led to significant depolarizations.^52^ But these effects were not present beyond the 2^nd^ post-natal week, suggesting a change in muscarine receptor effects. In these older motoneurons, the effects we report with muscarine were dominated by M2-/C-bouton-type effects of increased excitability, and these were similar in ChAT-K_V_2.1^OFF^ and K_V_2.1^ON^ motoneurons.

It therefore seems most likely that the differences in findings between the aforementioned studies and ours can be reconciled by considering the maturity of the preparations and the specificity of GxTX-1E for K_V_2 channels.

### A potential non-conducting role for K_V_2 channels at C-bouton synapses

Given that muscarine increased excitability in ChAT-K_V_2.1^OFF^ motoneurons despite the absence of K_V_2.1 channels, and our behavioral data showed that ChAT-K_V_2.1^OFF^ mice could normally amplify their motor output, it is likely that K_V_2.1 channels are not necessary for M2 receptor-mediated motor amplification by C-boutons. Our finding that K_V_2.2 channels are co-expressed with K_V_2.1 at C-bouton synapses suggests that if K_V_2 channels are important for neuronal function, K_V_2.2 can function independently of K_V_2.1 during behaviors requiring C-bouton activation.

We propose that K_V_2 channels have a minimally conducting role in mature motoneurons. That is, we suggest that in motoneurons, K_V_2 channels have a primarily non-canonical function.^27^^,^^30^^,^^32^^,^^33^^,^^53^^,^^54^^,^^55^^,^^56^

What role might K_V_2 channels play at C-bouton synapses? K_V_2 channels in the PM form physical links with the ER membrane via VAP proteins, creating tight EJPs.^9^^,^^28^^,^^29^^,^^30^ There is growing evidence in central neurons and smooth muscle suggesting that K_V_2.1 is responsible for the spatial and functional coupling of channels such as L-type calcium channels and RyRs into a Ca^2+^-signaling microdomain. Although it does not seem that RyRs are expressed at C-bouton synapses, there are several different proteins apposing C-boutons that are Ca^2+^-dependent (such as SK2/3), leading to the suggestion that K_V_2 channels may serve a role in the regulation of these proteins in motoneurons.^55^^,^^57^ Given that K_V_2.1 knockout did not have an effect on C-bouton function, it is unlikely that other C-bouton components were affected. We therefore suggest that K_V_2.2 channels serve a similar role and can subsume the non-conducting roles of Kv2.1. Further studies using double knock out experiments could determine the combined roles of K_V_2 channels in motoneuron and C-bouton physiology.

Evolution led to the development of C-bouton synapses to amplify motor output in a task-dependent manner.^3^^,^^4^ To understand the mechanisms of this important amplification, it is necessary to identify the structure and function of the plethora of proteins clustered in this region and to understand how these proteins interact with each other. We demonstrate that in motoneurons, post-synaptic K_V_2.1 and K_V_2.2 are co-expressed and likely serve predominantly non-conducting roles. However, the data also highlight that there is still much to learn regarding synaptic mechanisms of C-bouton motor amplification.

### Limitations of the study

Here, we targeted the largest motoneurons, reasoning that C-bouton amplification is principally evident in high-motor output tasks^4^ and is thus related to recruitment of the faster (and larger) motoneurons that innervate higher force-producing fast-twitch muscle fibers. In fast vs. slow mouse motoneurons, there are differences in the expression of channels that contribute to membrane voltage bistability, including Nav1.6, Trpm5, and K_V_1.2.^58^ Fast motoneurons have a higher density of C-boutons^59^; it is possible that there are clustering/expression differences in K_V_2 channels at these sites between slow and fast motoneurons, although none have been reported. In any case, taking the electrophysiological data together with the behavioral data together with the known high density of C-boutons on motoneurons, it is unlikely that a bias toward the larger motoneurons significantly impacted our conclusions.

## STAR★Methods

### Key resources table

**Table:** 

REAGENT or RESOURCE	SOURCE	IDENTIFIER
**Antibodies**		

VAChT	Millipore	Cat#ABN100; RRID:AB_2630394
Alexa Fluor® 647 donkey anti-goat	Thermo Fisher Scientific	Cat# A-21447; AB_2535864
Alexa Fluor® 555 donkey anti-rabbit	Thermo Fisher Scientific	Cat# A-31570; RRID: AB_2563181
Alexa Fluor® 555 goat anti-mouse IgG1	Thermo Fisher Scientific	Cat# A-21127; RRID:AB_141596
mouse anti- KV2.2 IgG1	UC Davis/NIH NeuroMab Facility	K37/8; RRID:AB_2750662
rabbit anti- KV2.1	Millipore	Cat# AB5186-200UL; RRID:AB_2131651
goat anti-choline acetyltransferase	Millipore	Cat# AB144P; RRID:AB_2079751
Alexa Fluor® 488 donkey anti-goat	Thermo Fisher Scientific	Cat# A-11055; RRID: AB_2534102
3% normal donkey serum	Millipore	SC30-100ML
Triton X-100	Millipore	648464

**Chemicals, peptides, and recombinant proteins**		

GxTX-1E	Tocris	cat#5676
Muscarine	Sigma	cat#M6532

**Deposited data**		

https://github.com/Brownstone-lab/KV2_paper_Allfiles_for_jupyterNB	Github	N/A

**Experimental models: Organisms/strains**		

Hb9:eGFP mice; B6.Cg-Tg(Hlxb9-GFP)1Tmj/J	JAX	stock no. 005029
ChAT cre mice; B6; 129S6-Chattm2(cre)Lowl/J	JAX	stock no. 006410
KCNB1(+/lox) mice	MRC Harwell	EMMA Strain ID: 08338
C57BL/6 mice	JAX	STRAIN CODE: 027

**Software and algorithms**		

FIJI image J	FIJI image J	RRID:SCR_002285
Microsoft Excel	Microsoft Corporation	RRID:SCR_016137
Anaconda package	Anaconda	RRID:SCR_018317
IMARIS	Bitplane	RRID:SCR_007370
Zen digital imaging	Zeiss	RRID:SCR_013672

**Other**		

Multiclamp 700A	Axon Instruments	RRID:SCR_021040
CED Power3 1401	CED Ltd	RRID:SCR_017282
P97 puller	Sutter Intruments	RRID:SCR_016842
Leica DMLFSA	Leica Microsystems	N/A
vibrating microtome Model 7000 smz-2	Campden Instruments Ltd	N/A
Vetbond	3M	No.1469SB
LSM 800 inverted confocal microscope	Zeiss	RRID:SCR_015963
Cryostat CM3050 S	Leica	RRID:SCR_016844
Grip strength test	Bioseb	N/A

### Resource availability

#### Lead contact

Further information and requests for resources and reagents should be directed to and will be fulfilled by the lead contact, Robert M. Brownstone (r.brownstone@ucl.ac.uk)

#### Materials availability

No new materials were created for this work.

#### Data and code availability

• Data: All data can be accessed from the following github repository: https://github.com/Brownstone-lab/KV2_paper_Allfiles_for_jupyterNB.
• Code: All code and Python analysis notebooks can be accessed from the following github repository: https://github.com/Brownstone-lab/KV2_paper_Allfiles_for_jupyterNB.
• Additional information: Any additional information required to reanalyse the data reported in this paper is available from the lead contact upon request.

### Experimental model and study participants

There are no human study participants.

#### Animals

All experiments were approved by the University College London Animal Welfare and Ethical Review Body, and performed under Project Licences 70/9098 and PP2688499 granted under the Home Office Animals (Scientific Procedures) Act (1986).

Three different mouse lines were used for this work. Wild type C57BL/6J were acquired from Charles River Laboratories, Inc (strain code: 632) and used for breeding transgenic and conditional knockout (cKO) lines, and for patch clamp electrophysiology experiments targeting cortical pyramidal neurons. Heterozygous *Kcnb1*^(+/lox)^ mice on a BL/6N background were acquired from the MRC Harwell facility (HA:006536; available through EMMA EM:08338), back-crossed to C57BL/6J mice, and bred to homozygosity (*Kcnb1*^(lox/lox)^). ChAT-IRES-Cre (ChAT^(+/Cre)^) mice were acquired from JAX (B6;129S6-Chattm2(cre)Lowl/J, stock no. 006410), and maintained on a C57BL/6J background. Hb9::eGFP mice, also maintained on a C57BL/6J background, were used for developmental immunohistochemistry (P2-21) and patch clamp experiments (P2-7). These mice were acquired from the Jessell lab in 2000 and are now available from JAX (B6.Cg-Tg(Hlxb9-GFP)1Tmj/J, stock no. 005029).

To generate *Kcnb1* cKO mice in which cholinergic neurons lack K_V_2.1 channels, *Kcnb1*^(lox/lox)^ mice were crossed with ChAT^(Cre/Cre)^ mice to produce ChAT^(+/Cre)^; *Kcnb1*^(+/lox)^ offspring. Cre-positive animals with a floxed allele were then crossed back to *Kcnb1*^(lox/lox)^ mice to produce ChAT^(+/Cre)^; *Kcnb1*^(lox/lox)^ offspring (Figure 1).

The number and sex of animals used for each experiment are declared in the figure legends.

#### Blinding and randomisation

For behavioural and anatomy experiments involving comparison of mature control and ChAT-K_V_2.1^OFF^ mice, animals were assigned to groups based on their genotype and sex, but experimenters remained blinded to group identity throughout all experiments. For electrophysiology experiments, blinding was not possible before recording, so this was done before data analysis. For developmental anatomy experiments, experimenters were blinded after imaging had taken place due to obvious anatomical differences in tissue of different ages. Sections of all ages were stained simultaneously in batches and well order was randomised.

### Method details

#### Immunohistochemistry

Animals were deeply anaesthetised by intraperitoneal injection of ketamine (100 mg kg^−1^) and xylazine (20 mg kg^−1^). Once insentient (loss of paw withdrawal), animals were perfused with 5 mL of phosphate buffered saline (PBS) followed by 20 mL of 4% paraformaldehyde. Vertebral columns were dissected and post-fixed for 24 h before spinal cords were dissected and cryoprotected in 30% sucrose for 72 h. Then, meninges were removed, and regions of interest segmented, frozen in OCT solution, and stored at −20°C. Transverse sections of 30–50 μm were cut using a cryostat (CM3050 S, Leica) and stored in 1x PBS as free floating sections.

To visualise motoneurons, C-boutons, and K_V_2.1 channels, sections were washed 3 times in PBS (10 min per wash) and then incubated for 1 h in blocking solution containing 3% normal donkey serum (NDS, SC30-100ML, Merk Millipore) and 0.2% Triton X-100 (648464, Merk Millipore) diluted in PBS (PBST). Sections were then incubated in blocking solution containing goat anti-choline acetyltransferase (ChAT, 1:250, Millipore Cat# AB144P; RRID:AB_2079751) and rabbit anti- K_V_2.1 (1:500, Millipore Cat# AB5186-200UL; RRID:AB_2131651) antibodies for 48 h at 4°C, then washed (3 × 10 min in 1xPBS) and incubated for 2 h at room temperature (22°C) in blocking solution containing the secondary antibodies Alexa Fluor 555 donkey anti-rabbit (1:500, Thermo Fisher Scientific Cat# A-31570, RRID: AB_2563181) and Alexa Fluor 488 donkey anti-goat (Thermo Fisher Scientific Cat# A-11055; RRID: AB_2534102, 1:500). Finally, sections were washed (3 × 10 min in 1xPBS) before being mounted on glass slides with Mowoil 4-88 (Carl Roth GmbH & Co. Kg).

To visualise C-boutons and K_V_2.2 channels, staining for goat anti-ChAT and mouse anti- K_V_2.2 IgG1 (UC Davis/NIH NeuroMab Facility, K37/89, RRID:AB_2750662, 1:200) were done sequentially (ChAT first then K_V_2.2) using the same incubation times as above for each step. The same primary, secondary, and blocking solutions and concentrations were used for ChAT staining. For K_V_2.2 channels, NDS was substituted for normal goat serum (NGS, 3%) and Alexa Fluor 555 goat anti-mouse IgG1 (Thermo Fisher Scientific Cat# A-21127; RRID:AB_141596, 1:500) was the secondary antibody.

To determine whether the knockout strategy was successful, control (females, animals = 3, slices = 3 per animal, age = P21) and cKO (females, animals = 3, Slices = 3 per animal, age = P21) slices stained for ChAT and K_V_2.1 were imaged using a Zeiss LSM 800 confocal microscope (Zeiss LSM 800 inverted confocal microscope with Airyscan, RRID:SCR_015963×), 20× objective (1 AU aperture) and Zeiss ZEN Blue Edition software (ZEN Digital Imaging for Light Microscopy, RRID:SCR_013672). Tile scan images of spinal cord hemi-sections were stitched and opened in Image J analysis software (RRID:SCR_002285) for processing. Using the particle analysis package, thresholding was performed on all images using the same settings to produce a black and white image showing only K_V_2.1 puncta. Regions of interest (ROI) of the same dimensions were defined to segment analyses of dorsal, intermediate, and ventral laminae. The nucleus counter function was then used to automatically produce a count of all puncta within the ROI, which was expressed as density (number per 100 μm^2^).

##### Postnatal development of K_V_2.1 and C-boutons on motoneurons

Procedures for assessing the postnatal development of C-boutons and K_V_2.1 channels on lumbar motoneurons were largely the same as those described above, with a few differences. Lumbar (L4-5) sections were cut (50 μm) from neonatal (P2-3), transition (P6-7) and motor mature (P21) Hb9::eGFP transgenic mouse spinal cords. Vesicular Acetylcholine Transferase polyclonal antibody (VAChT, 1:500, Millipore Cat# ABN100; RRID:AB_2630394) was used to visualize C-boutons and K_V_2.1 was visualised using the same antibody as described above. Secondary antibodies used were Alexa Fluor 647 donkey anti-goat (1:500, Thermo Fisher Scientific Cat# A-21447;AB_2535864) and Alexa Fluor 555 donkey anti-rabbit (1:500, Thermo Fisher Scientific Cat# A-31570;RRID: AB_2563181).

60x confocal z stack (0.4μm steps through tissue thickness) images were captured with the LSM 800 inverted confocal microscope and then pseudo-named in order to perform blinded analyses. Three-dimensional (3D) reconstructions of each motoneuron were then rendered using IMARIS Software (Bitplane, RRID:SCR_007370) using the following procedure. First, in the 3D isometric view, solid surfaces of the motoneuron soma (Hb9::eGFP signal), C-boutons, and K_V_2.1 were created using the rendering and thresholding tools. The masking feature was then used to select K_V_2.1 clusters within 1 μm of the motoneuron surface and VAChT^+^ C-boutons. IMARIS was used to generate volume and surface area data for each motoneuron and associated C-boutons and K_V_2.1 clusters, and these were exported to an Excel (Microsoft Corporation, 2018, RRID:SCR_016137) spreadsheet. Subsequent analyses were performed using python programming language run in Jupyter Labs environment. Cells were excluded only if quality of staining precluded accurate rendering of the cell.

Intensity plots were made in ImageJ by drawing a ROI with a polygon line around the perimeter of the cell, to connecting the centre points of all C-bouton puncta. The same ROI was copied to the K_V_2.1 channel and the relative intensities were measured (intensity/maximum intensity) for C-boutons and K_V_2.1. The relative intensity values (y axis) were then plotted against the corresponding distance values (x axis).

#### Patch clamp electrophysiology

##### Slice preparation

Current clamp experiments were performed as described in Smith and Brownstone (2020). Mice of all ages were administered an intraperitoneal bolus of ketamine (100 mg kg-1) and xylazine (20 mg kg-1) and decapitated following loss of hind-paw withdrawal. The vertebral column was quickly excised and pinned (ventral-side-up) to a silicone dish containing ice-cold (0°C–4°C) normal artificial cerebrospinal fluid (nACSF) saturated with 95% carbogen. nACSF was made in 18 MΩ water with the following (in mM): 113 NaCl, 3 KCL, 25 NaHCO3, 1NaH2PO4, 2 CaCl, 2 MgCl2 and 11 D-glucose, pH 7.4 (Mitra & Brownstone, 2012). A vertebrectomy was performed to reveal the spinal cord, which was stripped of dura matter and extracted from the vertebral column. The spinal cord was then glued (3M Vetbond, No.1469SB) ventral-side-up to a pre-cut block of agarose (∼8% in ddH_2_O) and mounted on a cutting chuck using superglue. These steps were completed as quickly as possible to ensure viability of slices from animals in the third post-natal week, typically within 3 min.

The chuck was transferred to the slicing chamber of a vibrating microtome (Model 7000 smz-2, Campden Instruments Ltd) containing ice-cold slicing solution made up of the following (in mM): 130 potassium gluconate, 15 KCL, 0.05 EGTA, 20 HEPES, 25 glucose, 3 kynurenic acid, pH 7.4.^60^^,^^61^ 350 μm slices were cut and transferred to the incubation chamber to rest in nACSF (32°C) for 30 min. The incubation chamber was then allowed to equilibrate to room temperature (maintained at 23°C) for at least 30 min before recording.

For voltage clamp experiments, animals were similarly anaesthetised and decapitated, and the spinal cord isolated, with oblique lumbar slices (350 μm thick) made as previously described,^62^ incubated at 37°C for 30-45min, and then maintained at room temperature (∼20°C) prior to being used for experiments.

##### Recording and analyses: Current clamp

Using a DMLFSA microscope (Leica DMLFSA; Leica Microsystems, Wetzlar, Germany), putative motoneurons were identified as the largest cells in the motor pools of the spinal cord. Patch pipettes were pulled using a P97 Flaming/Brown horizontal Micropipette Puller (Sutter Instrument, RRID:SCR_016842) to a resistance of 1.5–4 MΩ. Patch pipette electrodes were filled with an internal solution consisting of (in mM): 131 K-methanesulfonate, 6 NaCl, 0.1 CaCl2, 1.1 EGTA, 10 HEPES, 0.3 MgCl2, 3 ATP-Mg, 0.5 GTP-Na, 2.5 L glutathionine, 5 phosphocreatine, pH 7.25 adjusted with KOH, osmolarity 290–300 mOsm.

Recordings were made using a MultiClamp 700A amplifier (Axon Instruments, Inc), low pass filtered at 10 kHz and digitized at 25 kHz using a CED Power3 1401 (Cambridge Electronic Designs Limited). All experiments were performed in current clamp mode and data captured using Signal software (Cambridge Electronic Design Ltd, Cambridge, UK, RRID:SCR_017282). Once whole cell configuration was achieved, the bridge was balanced, and capacitance neutralized prior to commencing recording. Motoneurons were injected with a small negative rectangular pulse (500 ms duration) and the voltage responses of 15–30 traces were averaged to measure input resistance and whole-cell capacitance (WCC). Resistance was measured as the peak voltage change to the injected current and τ calculated from an exponential curve fitted to the response (automated in Signal). WCC was calculated using resistance and τ values and cross-checked against the values automatically recorded by the software during the experiment. Rheobase was defined as the minimum amount of current needed to evoke an action potential. Motoneuron frequency-current (ƒ-I) graphs were generated by injecting depolarizing current steps increasing from 0 nA until maximum firing was observed. The excitability of the cell (gain) was determined by measuring the slope of the main linear portion of ƒ-I plots for first interval frequency (instantaneous frequency of the first two spikes), and overall frequency (mean instantaneous frequency of all spike in a train). Action potential half widths (1/2 width), spike amplitude, and fast afterhyperpolarization (fAHP) were measured from 15 to 30 averaged single APs evoked with a 20 ms rectangular current pulse (to ensure stimulus artefact did not preclude measurement). The ½ width was calculated as the time between the 50% rise and 50% fall in amplitude of the AP. Spike height was measured as the voltage difference between the threshold (voltage at maximum positive value of the second derivative of membrane potential of AP) and the peak of the AP. The fAHP was measured as the difference between the voltage baseline and the most negative point on the first trough of the AP. Afterpotential measurements (mAHP amplitude and mAHP half decay time) were taken from averages of 15–30 single APs evoked with a 1 ms duration current pulse (to ensure stimulus artefact preceded mAHP). The mAHP amplitude was calculated from baseline to the most negative point on the trough. The mAHP half decay time is calculated as half the time taken (ms) from the most negative point of the mAHP to baseline. Cells were held at −65 mV for single evoked APs.

##### Recording and analysis: Voltage clamp

Motoneurons were visualized with a digital camera (Nikon, DS-Qi1Mc) using infrared differential interference contrast (DIC) optics on an Eclipse E600FN Nikon microscope (Nikon, Japan). Putative motoneurons were identified as above. For single electrode voltage-clamp recordings, a MultiClamp 700B or an Axopatch 200B amplifier (Molecular Devices, Sunnyvale) was used with signals filtered at 5 kHz and acquired at 50 kHz with a Digidata 1440A A/D board (Molecular Devices, Sunnyvale) and Clampex 10 software (Molecular Devices, Sunnyvale). A Flaming-Brown puller (P1000, Sutter Instruments, USA) was used to obtain borosilicate thick glass (GC150F, Harvard Apparatus, UK) pipettes, that were then microforged to a resistance of ∼1–3 MΩ using a MF2 Narishige Microforge (Narishige Group, Japan). For dual electrode recordings, an additional electrode connected to a ELC-03X amplifier (NPI Electronics) was used in current clamp mode as a voltage follower. Patch pipettes were backfilled with an intracellular solution containing (in mM) 125 K-gluconate, 6 KCl, 10 HEPES, 0.1 EGTA, 2 Mg-ATP, pH 7.3 with KOH, and osmolarity of 290–310 mOsm.

To study outward currents, 500ms-long voltage steps from −90mV up to +110mV were applied in +10mV increments (2s-long sweeps), in the present of CdCl_2_ (100μM) and tetrodotoxin (TTX, 0.5 μM) to reduce the effects of Ca^2+^ and Na^+^ channels activation. In some cases the amplitude of the largest step was adjusted accordingly to avoid damaging the cell, or the sweep length was increased to 5s or 10s. The current size was considered as the difference between the baseline and the amplitude of the last 20ms of the voltage step. To test the effect of GxTX-1E (100nM), estimated currents were scaled to the maximum current amplitude obtained prior to toxin administration (I_max control_), and the largest values compared.

Series resistance, input resistance and holding voltage (V_hold_ = −90mV) were monitored throughout the experimental procedure in order to track the quality of the recordings before and after applying toxin. We employed a -5mV step prior to the positive voltage steps for studying K^+^ currents, and from here the series resistance was calculated by dividing the voltage pulse by the amplitude of the observed transient peak current and input resistance by diving the voltage step by the amplitude of the stable state current. The currents measured from lumbar motoneurons were several nA in amplitude, so we only included experiments in which the series resistance and input resistance did not differ more than ∼25% before and after application of GxTX-1E. Initial uncompensated series resistance was typically low (∼2-6MΩ), and compensation by 40%–80% was used to control variations before and after drug application; if the uncompensated series resistance changed >50%, the recording was stopped to avoid unwanted voltage errors.^63^ Inclusion and exclusion of voltage clamp experiments can be seen at https://github.com/Brownstone-lab/KV2_paper_Allfiles_for_jupyterNB.

For dual patch recordings, a second electrode attached to the same motoneuron monitored the real voltage of the cell, which was compared with the voltage command from the voltage clamp patch electrode and the predicted voltage estimated using the series resistance and Ohm’s law as follows (2):

$$V_{predicted}=V_{pipette}\;-\left(R_{series}\times I_{feedback}\right)$$

in which V_predicted_ is the calculated voltage estimate when taking into account the voltage error, V_pipette_ is the voltage command from the voltage clamp electrode, R_series_ is the series resistance and I_feedback_ is the measured current that flows across the amplifier’s feedback resistor.

##### Mature motoneurons

For these experiments, the experimental unit (N) was considered to be individual motoneurons and is disclosed in the results section and figure legend (as well as animal number and sex) for each experiment. To avoid sampling γ-motoneurons we selected the largest motorneurons in motor pools with resting membrane properties consistent with those of verified ɑ-motoneurons- this was determined *a priori*. As a result, the majority of motoneurons sampled had input resistance values ≤50 MΩ. During analysis, we discovered a sampling bias between control and cKO motoneurons, whereby the control group had disproportionately more neurons with input resistance values >50 MΩ. To account for this we only included motoneurons with input resistance values ≤50 MΩ (Figure 3B).

##### Drugs and toxins

Baseline firing characteristics were recorded in nACSF for all experiments prior to the perfusion of drugs or toxins. To assess the effect of inhibiting K_V_2 channels, 100 nM GxTX-1E (Tocris, cat# 5676) in nACSF was perfused through the recording chamber for 10 min prior to the 2^nd^ recording.^20^^,^^34^^,^^38^ For experiments using muscarine (10μM, Sigma-Aldrich, cat# M6532), slices were perfused for 5 min prior to the 2^nd^ recording.^3^^,^^44^^,^^52^

##### Cortical pyramidal neuron patch clamp experiments

Coronal brain slices (350 μm) were made from wild type C57BL/6J male mice aged P14-15 (*N* = 3). Whole cell, current clamp recordings were made from visually identified pyramidal neurons in layer 5 of the cerebral cortex. Subsequent procedures were performed as described for mature motoneurons above.

#### EMG recording for C-bouton motor amplification

Bipolar, intramuscular electrodes were fabricated using materials and methods described in detail by.^64^

Procedures for surgical implantation were also based on those first described by.^64^ Mice were anaesthetised with isoflurane (5% with O_2_) and tested for loss of the paw withdrawal reflex before proceeding. Once insentient, the back and both hindlimbs were shaved, cleaned with 70% ethanol and then surgical iodine solution. An incision measuring the width of the EMG connector was made between the scapulae and another of similar size was made in the centre of the back at the level of the hips. Wires from the connector were tunnelled from the rostral to the caudal incision and the connector was secured in place using 4-0 sutures (ETHICON, W8683). Incisions were then made over the medial gastrocnemius and tibialis anterior of each hindlimb and corresponding wires tunnelled from the rostral incision to the muscle. Wires were inserted through the belly of the muscle up to the pre-made proximal knot in the wires and another distal knot was made to secure the recording sites in to muscle. The wires were trimmed, all incisions were washed with saline, and then closed with 7-0 sutures (ETHICON, W8702). Prior to withdrawing anaesthesia, the mouse was administered buprenorphine (Vetergesic, 0.1 mg/kg) and then transferred to a recover chamber maintained at 37°C until fully awake and ambulatory. Mice were given 1 drop of oral Metacam (Meloxicam, 1.5 mg/mL) analgesic daily for 5 days post-surgery, or as long as necessary.

After at least 10 full days of recovery, EMG recording sessions were undertaken. Immediately prior to recording, mice were lightly anaesthetised with isoflurane and the male connector was inserted into the female connector (sutured into the skin at the back of the neck). Once awake and ambulatory, mice were placed on a treadmill set to a speed of 0.15 m/s and recording commenced. Following walking experiments animals were transferred to a custom swimming pool (25°C) and muscle activity during swimming was recorded. Signals were amplified (x 1000) using an NL844 AC pre-amplifier (Digitimer Ltd) connected to an NL820 isolation amplifier (Digitimer Ltd), filtered (100 Hz-10KHz) and digitized with a Power 1401 interface and Spike2 software (Cambridge Electronic Design, Cambridge, United Kingdom).

#### Behavioural assessments and analysis

##### Running wheel experiments

Experimental mice were housed in large (rat) cages (Allentown, NexGen Rat 900) separated into 2 compartments with a Perspex divider, perforated to allow some social interaction. One compartment housed a companion mouse, that had no access to a running wheel and the other was occupied by either a control (ChAT^(w/w)^;*Kcnb1*^(f/f)^) or KO mouse (ChAT^(Cre/wt)^;*Kcnb1*^(f/f)^) which had *ad libitum* access to an externally mounted running wheel (Panlab, LE905). Mice underwent an initial acclimation period (7 days) in which they had no access to the running wheel. Following acclimation, mice had 24 h access to the wheels and data were collected (Panlab multicounter, LE3806) in either 5 or 1 min epochs for a total of 16 h from 19:00 to 11:00. Running distances were calculated from the circumference of the wheels (50.24 cm) and the number of revolutions. Experiments lasted 5 weeks in males and 6 weeks in females, after which data were exported to Microsoft Excel spreadsheets and analyzed using Python scripts in the Jupyter notebooks environment. Animals were excluded only if determined to be non-compliant. Non-compliance was determined as the mouse running less than 500 m per 16 h period. Only 1 mouse was excluded for non-compliance.

##### Treadmill speed and endurance experiments

Mice were acclimatised to the treadmill (Panlab, multilane treadmill, LE8710MTS) for a period of 3 days before testing: On day 1, the belt was kept static and mice were allowed to explore for 10 min. On day 2, the treadmill was set to a 15% incline and mice walked slowly (5 cm/s) for a total time of 15 min. The air puff encouragement was activated when contact was made with a grid at the rear of the treadmill. Day 3 consisted of a 20 min session consisting of 2 min at 5 cm/s followed by increments of 2 cm/s every 2 min. On maximum speed testing days, mice were placed in the treadmill and the speed increased by 3 cm/s every minute for 5 min to warm up. Then, the speed was increased by 5 cm/s every 20 s until mice were persistently lagging (i.e., when total air puff stimulation reached 10s). After 2 days recovery, mice were subjected to endurance tests involving the same 5 min warm up followed by up to 40 min (time cap) running at 60% of the maximum speed attained by individual mice. Again, the limit was determined as the time/distance at which mice received 10 s of air of stimulation.

##### Grip strength experiments

Grip strength was tested using a grip strength meter (Bioseb, France; Figure S5) over 3 days, with 2 days rest between each session. The experimenter held the tail of the mouse whilst supporting its weight on their other hand before lowering it onto the horizontal metal grid attached to the grip strength meter. Once the mouse gripped the bars, the experimenter slowly pulled backwards by the tail until the mouse released its grip. The peak force (grams) was recorded and the mouse was returned to its cage to rest for 1 min. This process was repeated 3 times for each mouse on each day of testing. Thus, mean force outputs (normalised to weight g/g body weight) for each animal were calculated from a total of 9 ‘pulls’.

### Quantification and statistical analysis

We center our analyses on estimation statistics because it focusses conclusions on magnitude, precision and biological significance of the results, thereby circumventing many of the flaws associated with null hypothesis significance testing (NHST).^65^^,^^66^^,^^67^^,^^68^ Hedges g effect sizes are calculated along with bootstrapped confidence intervals, which describe the range of effect sizes possible, rather than providing a single dichotomous decision (as done in NHST). Effect sizes can be classified as no effect (0–0.19), small (0.2–0.49), medium (0.5–0.79) and large (0.8; Hedges, 1981). Bootstrapped confidence intervals (CI) are used to determine the precision of the effect size; for confidence intervals that do not include 0, effect sizes are considered precise enough to attribute biological significance to the observed effects.

For unpaired comparisons of two groups, Gardner-Altman estimation plots were used, where the groups are plotted on the left axes and the Hedges g effect size with bootstrap resampled (5000 reshuffles) 95% confidence intervals plotted on the right. The Hedges g effect size is depicted as a dot; the 95% confidence interval is indicated by the vertical error bar. The values for mean difference (μ), effect size (es) and 95% confidence intervals [lower, upper] are displayed each plot. Experimental units (N) are described in the figure legends.

For paired experiments, Cumming paired estimation plots were used where the experimental units are plotted on the upper graphs and each paired set of observations is connected by a line. On the lower plots, effect sizes (Hedges g) are plotted with bootstrap resampled (5000 reshuffles) 95% confidence intervals. Effect sizes are depicted as dots; 95% confidence intervals are indicated by the vertical error bars. The bootstrapped mean differences (μ) are shown on the upper plots, and the effect sizes (es) and 95% confidence intervals [lower, upper] are displayed on the lower plots.

For IHC experiments assessing C-bouton and K_V_2.1 channel expression and clustering during development, 3 animals (male) were used for each age group from which 3 sections and 16-18 motoneurons (maximum 6 per slice) were sampled. Because we were able to sample the entire motoneuron soma for puncta density measurements, we consider the motoneuron to be the experimental unit in these experiments.

For analysis of EMG data, signals acquired during walking and swimming were converted to RMS amplitudes (τ = 40 ms) and the peak values of each burst for each muscle were averaged. Motor amplification ratio was expressed as mean RMS amplitude during swimming/walking. Mean amplitudes across muscles (MG or TA) were treated as individual experimental units. For wheel running experiments assessing distance run per day, repeated measured ANOVAs were used as a suitable alternative was not available for estimation statistics.

All figures were created using Seaborn v.0.10.0, DABEST^69^ and Matplotlib v.3.1.3 in the Python environment.
