# Supplementary material for: Kv2 channels do not function as canonical delayed rectifiers in spinal motoneurons
Source: iScience. 2024 Jul 3;27(8):110444. doi: 10.1016/j.isci.2024.110444 (PMC11325356; doi:10.1016/j.isci.2024.110444)
Supplement: Document S1. Figures S1–S6 [file mmc1.pdf]

iScience, Volume *27*

Supplemental information

Kv2 channels do not function as canonical delayed rectifiers in spinal motoneurons

Calvin C. Smith, Filipe Nascimento, M. Görkem Özyurt, Marco Beato, and Robert M. Brownstone

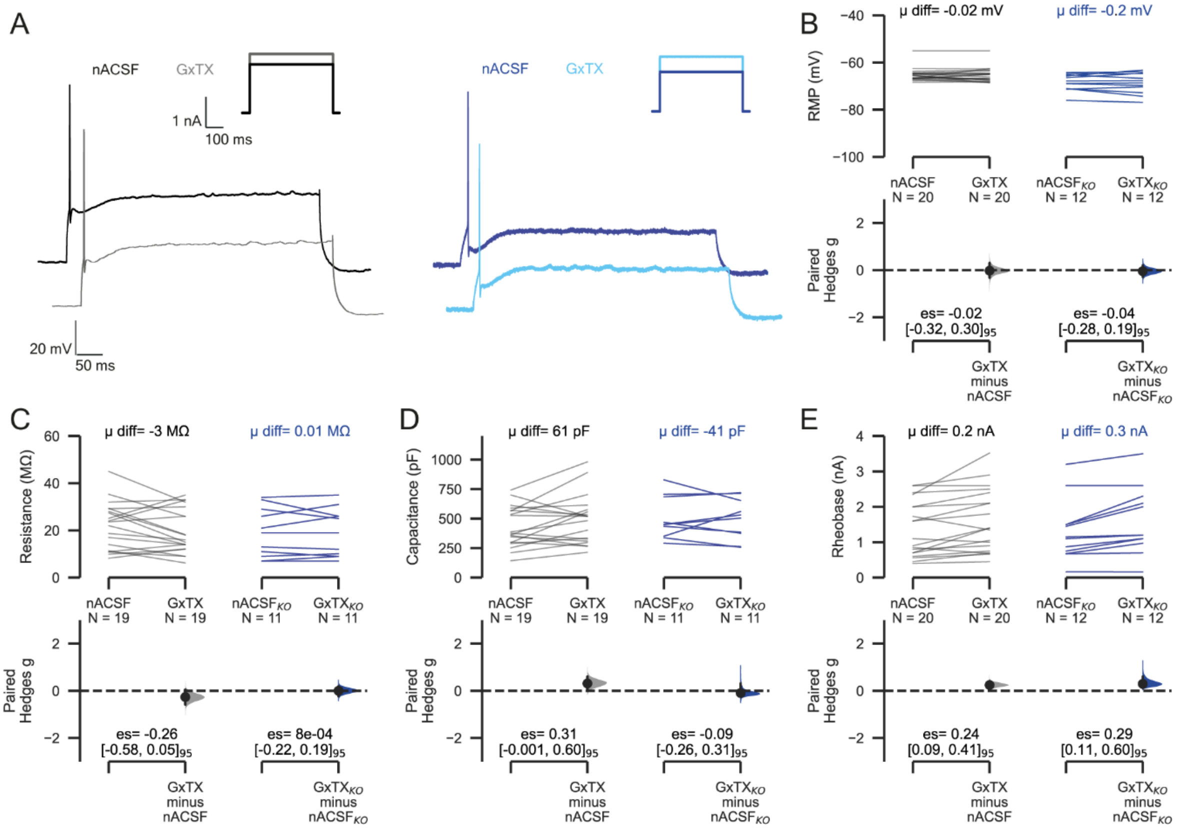

Figure S1. Related to Figure 3. The effect of GxTX-1E on passive membrane properties. (A) Representative traces from control and ChAT-K$_V$2.1$^{\mathbf{OFF}}$ motoneurons depicting a slight increase in rheobase following perfusion with 100 nM GxTX-1E. **(B-E)** Paired Hedges g for control (left) and ChAT-K$_V$2.1^{OFF} (right) motoneurons in Cumming paired estimation plots, showing resting membrane potential **(RMP, B)**, input resistance **(C)**, whole cell capacitance **(D)**, and rheobase changes in response to toxin **(E)**. Experimental unit (N) = motoneurons. Number of animals used was as follows: control= 13 (6 females, 7 males), ChAT-K$_V$2.1$^{\mathbf{OFF}}$ **=** 6 (4 females, 2 males). Mean difference is abbreviated to “μ diff”, and hedges g estimation statistic is represented by “es”.

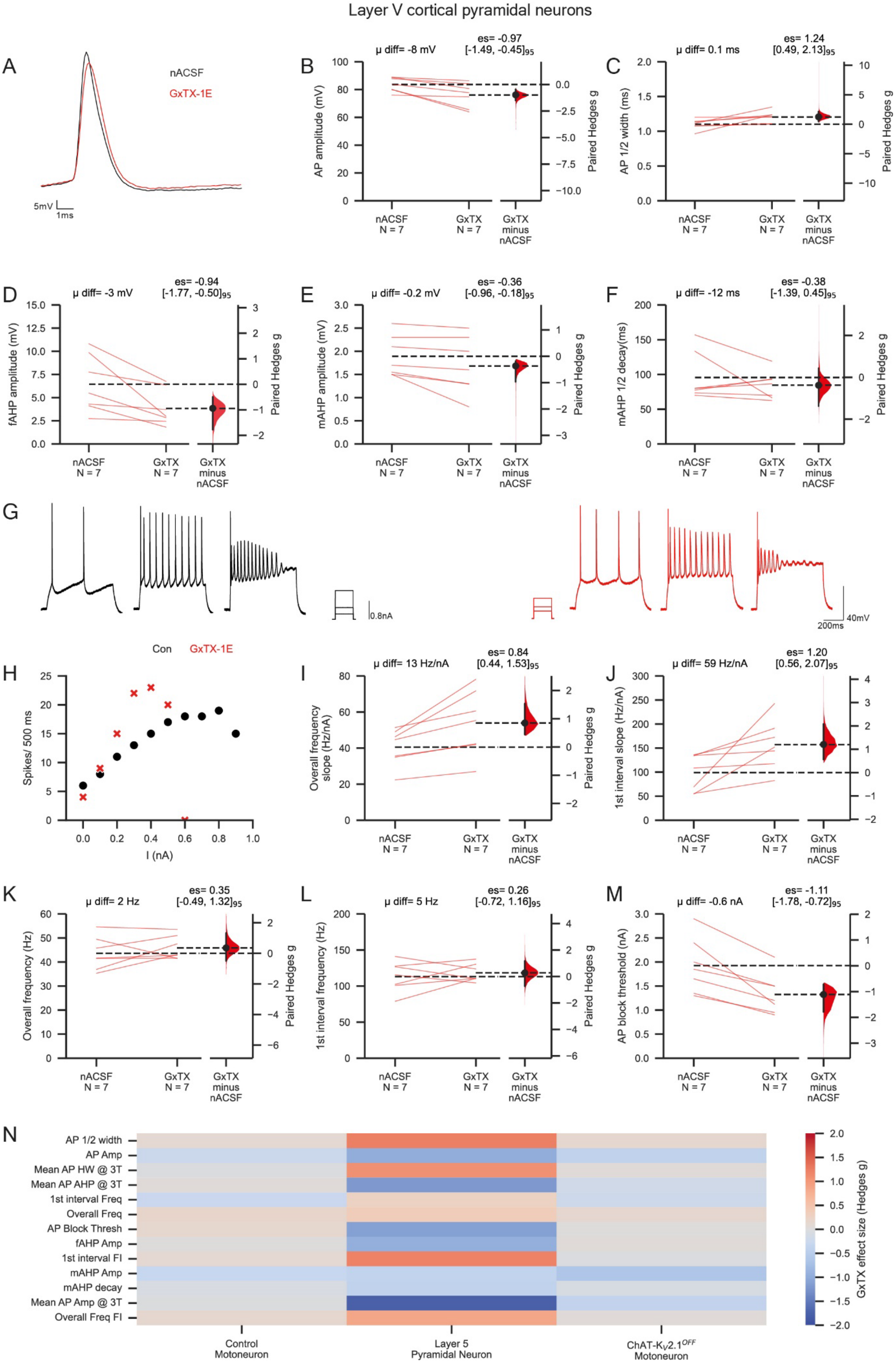

Layer V cortical pyramidal neurons
A
nACSF
GxTX-1E
5mV
1ms
B
μ diff= -8 mV
es= -0.97
[-1.49, -0.45]95
AP amplitude (mV)
Paired Hedges g
C
μ diff= 0.1 ms
es= 1.24
[0.49, 2.13]95
AP 1/2 width (ms)
D
μ diff= -3 mV
es= -0.94
[-1.77, -0.50]95
fAHP amplitude (mV)
E
μ diff= -0.2 mV
es= -0.36
[-0.96, -0.18]95
mAHP amplitude (mV)
F
μ diff= -12 ms
es= -0.38
[-1.39, 0.45]95
mAHP 1/2 decay(ms)
nACSF N = 7
GxTX N = 7
GxTX minus nACSF
G
0.8nA
40mV
200ms
H
Con
GxTX-1E
Spikes/ 500 ms
I (nA)
I
μ diff= 13 Hz/nA
es= 0.84
[0.44, 1.53]95
Overall frequency slope (Hz/nA)
J
μ diff= 59 Hz/nA
es= 1.20
[0.56, 2.07]95
1st interval slope (Hz/nA)
K
μ diff= 2 Hz
es= 0.35
[-0.49, 1.32]95
Overall frequency (Hz)
L
μ diff= 5 Hz
es= 0.26
[-0.72, 1.16]95
1st interval frequency (Hz)
M
μ diff= -0.6 nA
es= -1.11
[-1.78, -0.72]95
AP block threshold (nA)
N
AP 1/2 width
AP Amp
Mean AP HW @ 3T
Mean AP AHP @ 3T
1st interval Freq
Overall Freq
AP Block Thresh
fAHP Amp
1st interval FI
mAHP Amp
mAHP decay
Mean AP Amp @ 3T
Overall Freq FI
Control Motoneuron
Layer 5 Pyramidal Neuron
ChAT-Kv2.1OFF Motoneuron
GxTX effect size (Hedges g)

Figure S2. Related to Figure 3. GxTX-1E increases excitability of cortical layer V pyramidal neurons. (A) Representative single AP traces from layer V cortical pyramidal neurons showing the difference in spike morphology pre (nACSF, black) and post 10 minute perfusion with 100 nM GxTX-1E (red). **(B-F)** Cumming paired estimation plots for individual neurons plotted on the left axes with each paired set of observations (nACSF followed by 100 nM GxTX-1E) connected by a line. On the right axes, effect sizes (Hedges g) are plotted as a bootstrapped sampling distribution (5000 reshuffles). Mean effect sizes are depicted as dots; 95% confidence intervals are indicated by the vertical error bars. **(B)** shows the spike amplitude, **(C)** is action potential ½ width, **(D)** is the fAHP amplitude, **(E)** is the mAHP amplitude, and **(F)** is the mAHP ½ decay time. Experimental unit (N) = neurons recorded from 3 animals (2 males, 1 female). **(G)** Representative traces showing pyramidal neuron firing in response to 500 ms current steps of increasing magnitude before (nACSF, black) and after **(**GxTX-1E, red) GxTX-1E perfusion. **(H)** Scatter plot of number of spikes in response to increasing current input in a representative pyramidal neuron in nACSF (black circles) and after GxTX-1E perfusion (red crosses). **(I-M)** Cumming paired estimation plots for individual neurons plotted on the left axes with each paired set of observations (nACSF followed by 100 nM GxTX-1E) connected by a line. On the right axes, effect sizes (Hedges g) are plotted as a bootstrapped sampling distribution (5000 reshuffles). Mean effect sizes are depicted as dots; 95% confidence intervals are indicated by the vertical error bars. Plots show overall frequency slope **(I)**, 1st interval frequency slope **(J)**, maximum overall frequency **(K)**, maximum 1st interval frequency **(L)**, and AP block threshold **(M).** Experimental unit (N) = neurons recorded from 3 animals (2 males, 1 female). **(N)** Heat map showing the effect sizes (Hedges g) for GxTX-1E induced changes in action potential and firing characteristics in mature control motoneurons, layer 5 cortical pyramidal neurons, and mature ChAT-$K_V2.1^{OFF}$ motoneurons. Dark colours represent large positive (red) or negative (blue) effect sizes. Mean difference is abbreviated to "μ diff", and hedges g estimation statistic is represented by "es".

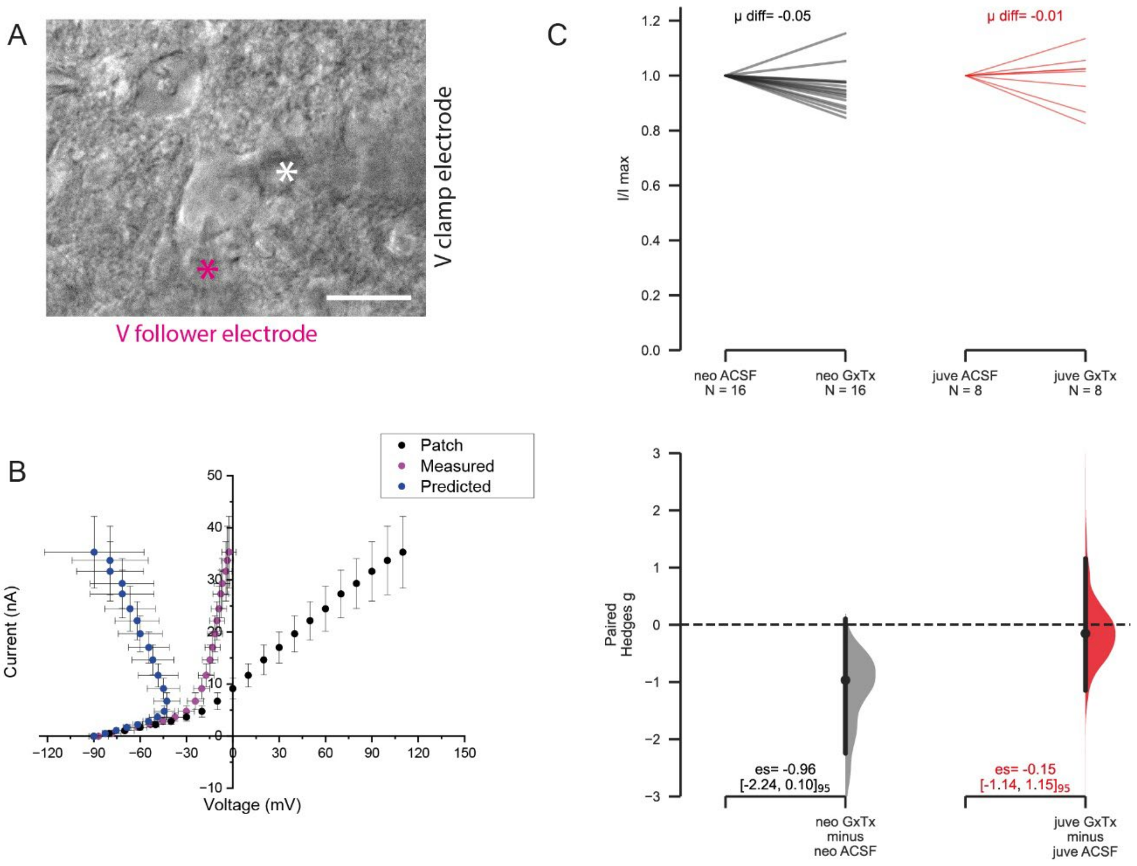

Figure S3. Related to Figure 4. Dual electrode voltage clamp recordings and voltage clamp study of K^+ currents. (A) 40x DIC image of a neonatal mouse lumbar motoneuron (P5) used for dual electrode experiments, with the position of the voltage clamp (white *) and voltage follower (lilac *) electrodes shown; **(B)** Example traces showing voltage clamp electrode outward current (left) and command voltage (right, black) overlaid with measured voltage obtained via the voltage follower electrode (right, lilac). Voltage steps are 500ms duration, and from -90mV up to +110mV; **(C)** Plot illustrating the mean (±SD) currents plotted against the command (black) and measured (lilac) voltages (n=8 motoneurons); **(D)** Cumming estimation plots for both neonatal and early juvenile mice, illustrating the effect of GxT1-E (100nM) on maximum outward currents obtained following increasing voltage steps (top panel), with respective bootstrapped paired Hedges g, and 95% confidence interval (bottom panel). SD – standard deviation. Mean difference is abbreviated to "μ diff", and hedges g estimation statistic is represented by "es".

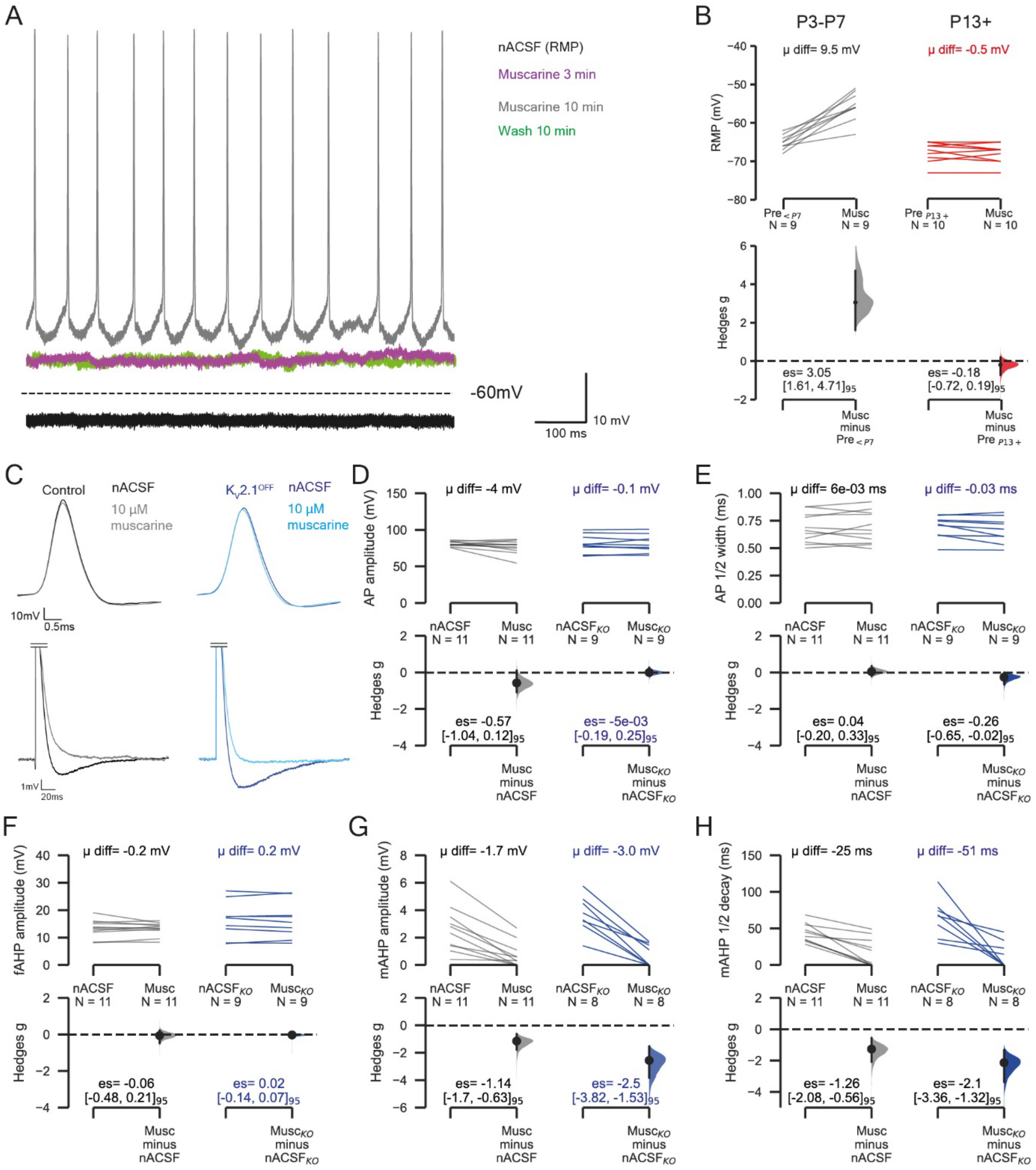

Figure S4. Related to Figure 5. Muscarine depolarises early post-natal motoneurons only and decreases mAHP in both control and ChAT-K_V2.1^{OFF} motoneurons.

(A) Traces from a P7 motoneuron showing that 10 µM muscarine induces large depolarisations, and sometimes AP firing in young motoneurons. No significant depolarisations were reported in motoneurons from animals older than P13. **(B)** Paired Hedges g for young (P2-P7, left) and mature (P13+, right) motoneurons are shown in Cumming paired estimation plots. Animal numbers were as follows: P2-3 = 6 female (8 MNs), 1 male (2 MNs); P13-21 = 4 females (8 MNs), 2 males (2 MNs). **(C)** Representative traces showing the effect of 10 µM muscarine on action potential spike (upper panels) and mAHP morphology (lower panels) in control (left) and ChAT-K_V2.1^{OFF} motoneurons (right). Double line shows spike truncation for visual purposes. **(D-H)** Paired Hedges g for control (left) and ChAT-K_V2.1^{OFF} (right) motoneurons are shown in Cumming paired estimation plots. Individual motoneurons are plotted on the upper graphs; each paired

set of observations (nACSF followed by 10 µM Muscarine) connected by a line. **(D)** shows the spike amplitude, **(E)** is action potential ½ width, **(F)** the fAHP amplitude, **(G)** is the mAHP amplitude, and **(H)** is the mAHP ½ decay time. Experimental unit (N) = motoneurons. Number of animals used was as follows: control= 8 (2 females, 6 males), ChAT-K_V2.1^{OFF} = 6 (2 females, 4 males). Mean difference is abbreviated to "µ diff", and hedges g estimation statistic is represented by "es".

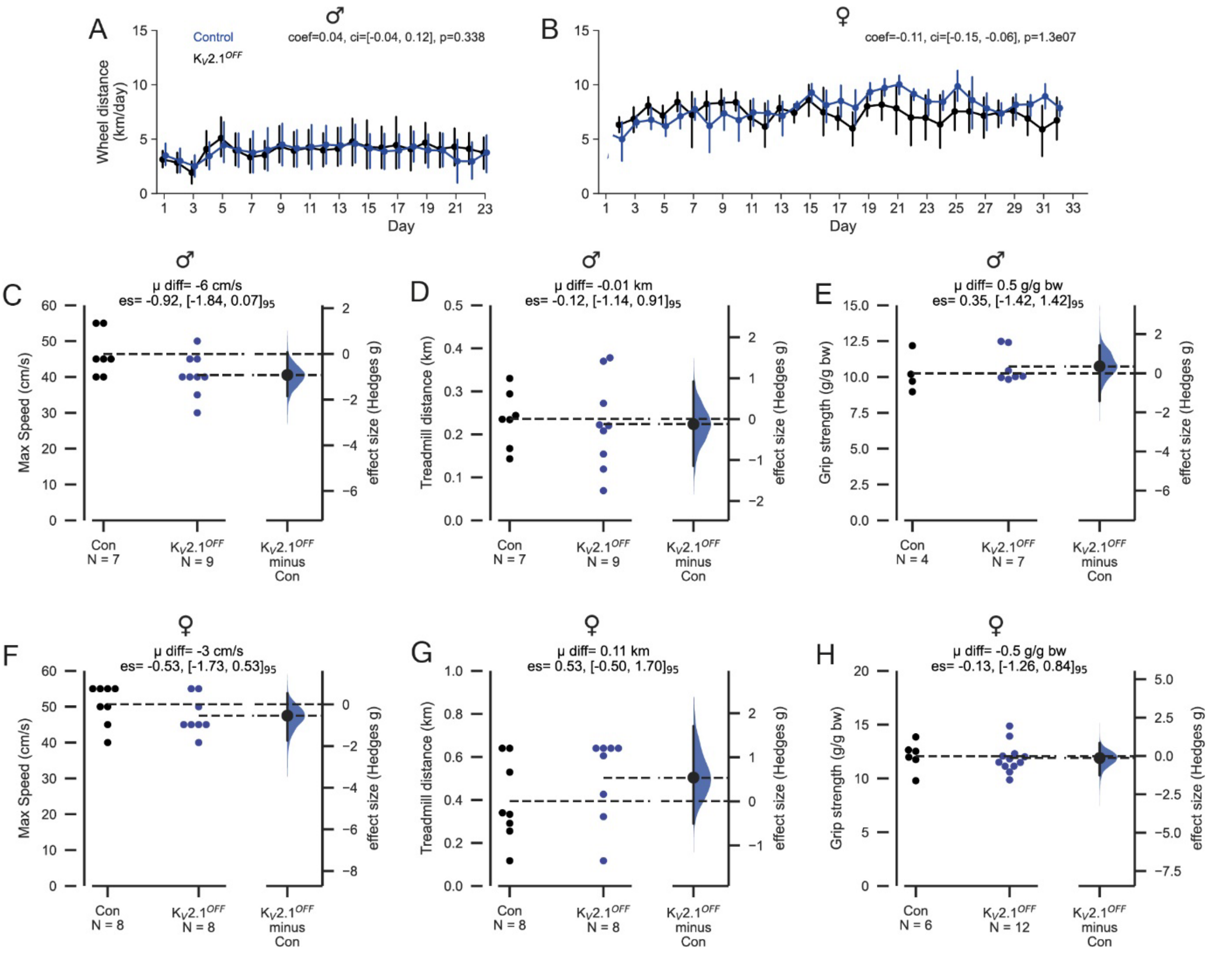

Figure S5. Related to Figure 6. Volitional activity and force output is not affected in ChAT-$K_V2.1^{OFF}$ mice .

(A-B) Distance run per day for male **(A)** and female **(B)** control (black; male N=6, female N=5) and ChAT-$K_V2.1^{OFF}$ (grey; male N=6, female N=6) mice. Output of repeated measures ANOVA shown at the top of each graph. Gardner-Altman estimation plots show treadmill and grip strength data in **(C-H)**. Maximum speed **(C)** and distance **(D)** run by male mice on a treadmill at 15° incline. **(E)** Grip strength (g) in male mice normalised to body weight (g). **(F-H)** as in **C-E**, but for female mice. Experimental unit (N) = animals. Mean difference is abbreviated to "μ diff", and hedges g estimation statistic is represented by "es".

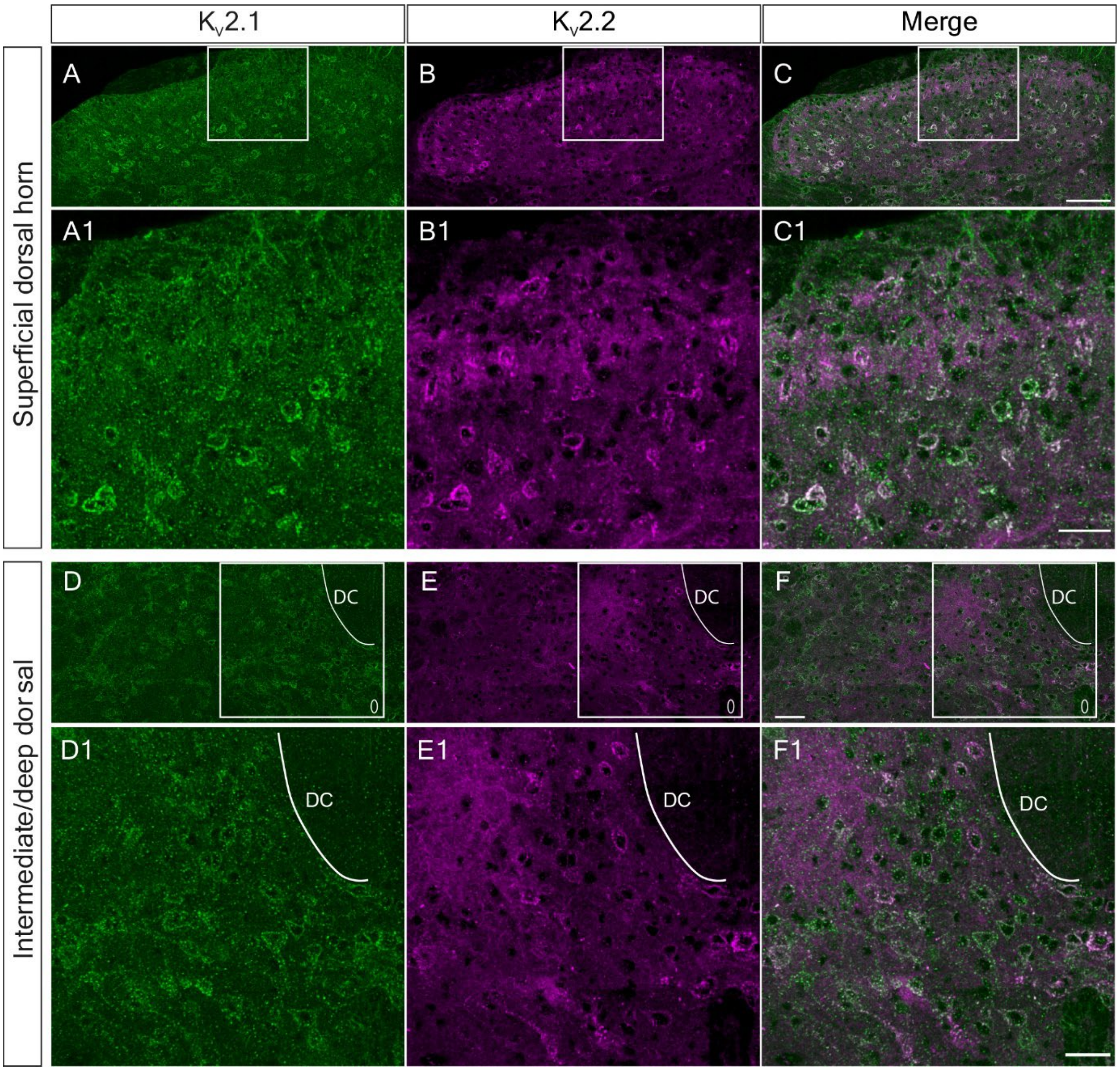

Figure S6. Related to Figure 7. K$_V$2 expression throughout the spinal laminae. (A-F1) 40x confocal tiled Z stack (5 x 1µm slices) projection images of the superficial and deep dorsal/intermediate laminae of the lumbar spinal cord stained for K$_V$2.1 **(A-A1, D-D1)** and K$_V$2.2 **(B-B1, E-E1)**; merged image shown in **(C-C1, F-F1)**. **(A-C)** Shows the superficial laminae of the dorsal horn, with (A1-C1) showing the region marked by a box in (A-C) cropped and expanded. (**D-F)** Shows the deep dorsal and intermediate laminae, with (D1-F1) showing the region marked by a box in (D-F) cropped and expanded. DC= dorsal columns, CC= central canals. Scale bars in (A-C) = 40 µm, (A1-C1) = 25 µm, (D-F) = 40 µm, (D1-F1) = 20 µm.
